# An efficient protocol for the synthesis of pyridines and hydroquinolones using IRMOF-3/GO/CuFe_2_O_4_ composite as a magnetically separable heterogeneous catalyst

**DOI:** 10.1038/s41598-023-36115-2

**Published:** 2023-06-05

**Authors:** Mohammad Ali Ghasemzadeh, Boshra Mirhosseini-Eshkevari, Jaber Dadashi

**Affiliations:** 1 Department of Chemistry, Qom Branch, Islamic Azad University, Post Box, Qom, 37491-13191 Iran; 2 Catalysts and Organic Synthesis Research Laboratory, Department of Chemistry, Iran University of Science and Technology, Tehran, 16846-13114 Iran

**Keywords:** Chemistry, Nanoscience and technology

## Abstract

This study reports a facile and cost-effective technique for preparing magnetic copper ferrite nanoparticles supported on IRMOF-3/GO [IRMOF-3/GO/CuFe_2_O_4_]. The synthesized IRMOF-3/GO/CuFe_2_O_4_ was characterized with IR, SEM, TGA, XRD, BET, EDX, VSM, and elemental mapping. The prepared catalyst revealed higher catalytic behavior in synthesizing heterocyclic compounds through a one-pot reaction between various aromatic aldehydes, diverse primary amines, malononitrile, and dimedone under ultrasound irradiations. Among the notable features of this technique are higher efficiency, easy recovery from the reaction mixture, removal of a heterogeneous catalyst, and uncomplicated route. In this catalytic system, the activity level was almost constant after various stages of reuse and recovery.

## Introduction

Graphene oxide (GO) is a compound with surface or edge functional groups and superior chemical structures and features, making it an excellent support for various nanostructures^1–4^. As graphene oxide has oxygen atoms in diverse isomers such as epoxide, carbonyl, carboxyl, and hydroxyl on its basal planes, various NPs can interact with GO sheets through charge-transfer interactions, physisorption, or electrostatic binding. This biodegradable nanostructure helps enhance the bioavailability of the nanocomposites and has some other advantages like high levels and thermal stability. GO acts as an anchoring support enabling uniform dispersion of the nanocomposites^5^.

In the last decade, nanoporous metal–organic frameworks (MOFs) comprising organic linkers and inorganic building units have been the subject of intense research^6^. This novel hybrid material includes a crystalline two or three and 1D open framework^7–9^. MOF-based structures have unique features such as structural diversity, high porosity, high pore volume, and higher capacity for guest molecular acceptance^10^. MOFs are used in various fields like separation and storage of gases and as a catalyst^11–13^. MOFs represent higher flexibility for designing catalytic centers than purely inorganic porous materials^14^. This flexibility makes them attractive substances for heterogeneous catalysts. There are several methods to modulate MOFs owing to the significant variety of metal ions and organic linkers. The appearance and size of the framework can be altered via different linkers and altering their connectivity by adding various functional groups on the linkers^15,16^. Using metal–organic frameworks as green catalysts accelerates the organic reactions. As mentioned in the previous authoritative articles, the metal ions in the structure of MOFs act as a Lewis acid and the carboxylate anion as a Lewis base. In this respect, the IRMOF-3 structure has been used as a bifunctional catalyst in the IRMOF-3/GO/CuFe_2_O_4_ structure due to the presence of metal cation sites (Zn^2^^+^) and also carboxylate anions^17^. The existence of non-coordinated amino groups structure in IRMOF-3 demonstrates that the basicity of the aniline-like amino group is enhanced when incorporated inside the pores of MOF channels.

Using multi-component reactions (MCRs) as an organized and efficient method has caused a revolution in designing novel approaches and creating multifarious chemical libraries with molecular diversity in the last decade. They have specific features such as higher atomic economy, straightforward reaction model, shorter reaction time, and conformity with other green chemistry principles. Hence, these chemical reactions have become the most potent and prevailing approach to synthesizing heterocyclic compounds^18^. Recently, numerous studies have been conducted on developing novel synthetic techniques to synthesize quinoline and pyridine compounds. This high attention is attributed to their promising and notable diversity in pharmacological and therapeutic activities like geroprotective, antitubercular, anticancer, antidiabetic, and analgesic activities^19^. Different developed synthetic approaches and catalysts, such as LiBr, CTAB, CuBr, salicylic acid, *L*-proline, and GO nanoparticles^20–25^, have been proposed to synthesize these biological and chemical molecules. However, they mostly encounter some disadvantages and negative points such as applying impotent and inefficient catalysts, low yield of the products, difficult reaction conditions, and tedious work-up procedures. Thus, designing and providing novel synthetic methods for these biological molecules is challenging.

**Figure 1:**
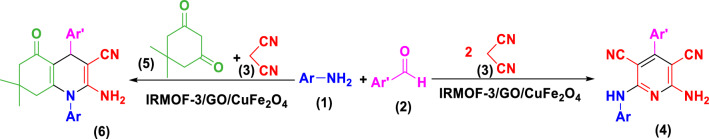
Synthesis of pyridines and quinolines using IRMOF-3/GO/CuFe_2_O_4_.

The present study aims to enhance more effective synthetic procedures, lower the number of isolated reaction stages, and minimalize wastes by preparing the heterocyclic compounds^26–28^. Next, we report a new and mild route for synthesizing heterocyclic compounds through a one-pot reaction between various aromatic aldehydes, diverse primary amines, malononitrile, and dimedone under ultrasound irradiations in the attendance of IRMOF-3/GO/CuFe_2_O_4_ nanocatalyst (Fig. 1).

## Experimental

### Preparing CuFe_2_O_4_ nanoparticles

A solution of FeCl_2_.4H_2_O and CuCl_2_.2H_2_O (molar ratio, 2:1) in deionized water (30 mL) was added to a concentrated solution of NaOH to reach a pH of 13. Then, the mixture was stirred vigorously at 70 °C until to formation of a black residue, including CuFe_2_O_4_. The precipitate was rinsed several times after cooling and dried overnight at 80 °C. Finally, it was calcinated for 5 h in a furnace at 700 °C.

### Preparation of IRMOF-3/CuFe_2_O_4_ nanocomposite

A mixture of CuFe_2_O_4_ (0.05 g), Zn (NO_3_)_2·_6H_2_O (0.066 g) and NH_2_-BDC (0.016 g) was dissolved in DMF (10 mL) under vigorous stirring. Next, the solution was transferred to an autoclave and heated at 120 °C for 10 h. The precipitate was separated after cooling, by a simple filtration. The obtained crystals were then soaked in 20 mL of DMF at 80 °C for 12 h. Lastly, the obtained IRMOF-3/CuFe_2_O_4_ was dried for 24 h at 40 °C^29^.

### Synthesis of IRMOF-3/GO/CuFe_2_O_4_ nanocatalyst

In this research, our catalyst including IRMOF-3/GO/CuFe_2_O_4_ was successfully prepared according to the modified Hummer's method and our previously published paper^30,31^, (See Supplementary Information, Fig. S1).

### General synthesis of 2-amino-4-aryl-6-substituted pyridine-3,5-dicarbonitrile (4a-r) using IRMOF-3/GO/CuFe_2_O_4_ nanocatalyst

IRMOF-3/GO/CuFe_2_O_4_ nanocomposite (0.003 g) was added to a solution of aromatic amine (1 mmol), malononitrile (2 mmol), and aldehyde (1 mmol) in 5 mL of EtOH. The mixture was sonicated under ultrasonic irradiations (25 kHz frequency) for 10–20 min. After completing the reaction, the obtained solid was filtered, and the contents were dissolved in acetone, and the insoluble IRMOF-3/GO/CuFe_2_O_4_ nanocomposite was separated by centrifuge. In the last step, the solvent was evaporated under a vacuum, and the precipitate was recrystallized using ethanol to provide the corresponding 2-amino-4-aryl-6-substituted pyridine-3,5-dicarbonitrile.

### General synthesis of hydroquinoline-3-carbonitrile derivatives (6a-d) in the presence of IRMOF-3/GO/CuFe_2_O_4_ nanostructure

A mixture of anilines (1 mmol), malononitrile (1.1 mmol), aldehyde (1 mmol), dimedone (2 mmol), and IRMOF-3/GO/CuFe_2_O_4_ (0.005 g) was added to a flask containing ethanol (5 mL). The mixture was sonicated for the appropriate times. The catalyst was separated over completion (monitored by TLC), by dissolving the obtained heterocyclic compounds in dichloromethane. Finally, the corresponding products in high purity were collected by evaporating the dichloromethane.

The scanned original spectral data of new compounds are provided in Supporting Information.

#### 2-amino-4-(4-cyanophenyl)-6-((4-methoxyphenyl) amino) pyridine-3,5-dicarbonitrile 4j

Yellow solid; m.p. 223–225 °C. IR spectrum ν, cm^−1^: 3365, 3172, 3051, 2932, 2370, 1705,1674, 1591, 1458, 1205; ^1^H NMR (250 MHz, DMSO-*d*_6_): 3.84 (s, 3H, CH_3_), 5.43 (s, 2 H, NH_2_), 6.76–7.04 (d, 2H, *J* = 8.2 Hz, ArH), 7.28–7.36 (d, 2H, *J* = 8.3 Hz, ArH), 7.65–7.75 (m, 4H, ArH), 9.85 (s, 1H, NH); ^13^C NMR (62.9 MHz, DMSO-*d*_6_) δ: 27.33, 31.39, 68.62, 76.82, 87.26, 101.03, 112.53, 123.45, 131.34, 133.43, 134.25, 138.56, 144.43, 146.33, 148.62, 156.47, 165.38, 168.28, 170.21, 183.31, 188.22; Anal. Calcd. For: C_21_H_14_N_6_O: C 68.84, H 3.85, N 22.94. O 4.37. Found: C 76.88, H 3.82, N 22.92 O 4.35; MS (EI) (m/z): 366.12 (M^+^).

#### 2-amino-6-((4-methoxyphenyl)amino)-4-(4-(methylthio)phenyl)pyridine-3,5 dicarbonitrile 4k

Yellow solid; m.p. 210–212 °C. IR spectrum ν, cm^–1^: 3363, 3170, 2555, 21,381, 1701, 1678, 1593, 1458, 1377, 1207; ^1^H NMR (250 MHz, DMSO-*d*_6_): 2.36 (s, 3H, CH_3_), 3.72 (s, 3H, CH_3_), 5.48 (s, 2 H, NH_2_), 6.76–7.04 (d, 2H, *J* = 8.2 Hz, ArH), 7.27–7.36 (d, 2H, *J* = 8.3 Hz, ArH), 7.65–7.78 (m, 4H, ArH), 9.82 (s, 1H, NH); ^13^C NMR (62.9 MHz, DMSO-*d*_6_) δ: 28.39, 32.33, 67.62, 77.82, 86.26, 102.03, 113.52, 125.44, 132.34, 134.43, 136.26, 139.56, 146.44, 148.33, 149.69, 154.47, 162.34, 164.28, 171.21, 181.32, 187.21; Anal. Calcd. For: C_21_H_17_N_5_OS: C 65.10, H 4.42, N 18.08. O 4.13, S 8.27. Found: C 65.14, H 4.45, N 18.04, O 4.316, S 8.25.; MS (EI) (m/z): 387.12 (M^+^).

#### 2-amino-4-(4-cyanophenyl)-1-(4-methoxyphenyl)-7,7-dimethyl-5-oxo-1,4,5,6,7,8 hexahydroquinoline-3-carbonitrile 6c

Yellow solid; m.p. 242–243 °C. IR spectrum ν, cm^–1^: 3321, 3062, 2985, 2233, 1608, 1716, 1678, 1381, 1284; ^1^H NMR (250 MHz, DMSO-*d*_6_) δ: 0.82 (s, 3H, CH_3_), 0.95 (s, 3H, CH_3_), 2.29 (d, 2H, *J* = 8.4 Hz, 2CH), 2.44 (d, 2H, *J* = 8.2 Hz, 2CH), 3.82 (s, 3H, CH_3_), 4.33 (s, 1 H, CH), 5.33 (s, 2 H, NH_2_), 6.74–7.05 (d, 2H, *J* = 8.6 Hz, ArH), 7.28–7.32 (d, 2H, *J* = 8.3 Hz, ArH), 7.62–7.78 (m, 4H, ArH); ^13^C NMR (62.9 MHz, DMSO-*d*_6_) δ: 28.33, 30.79, 78.61, 88.62, 92.24, 102.06, 117.46, 118.41, 124.54, 128.61, 130.54, 132.93, 133.22, 137.51, 142.63, 144.01, 149.32, 159.27, 166.88, 173.46, 178.81, 180.74, 183.31, 192.12, 194.81, 196.71; Anal. Calcd. For: C_26_H_24_N_4_O_2_: C 73.56, H 5.70, N 13.20, O 7.54. Found: C 73.54, H 5.73, N 13.24, O 7.52; MS (EI) (m/z): 424.19 (M^+^).

### 2-amino-1-(4-methoxyphenyl)-7,7-dimethyl-4-(4-(methylthio)phenyl)-5-oxo-1,4,5,6,7,8 hexahydroquinoline-3-carbonitrile 6d

Yellow solid; m.p. 256–258 °C. IR spectrum ν, cm^–1^: 3352, 3186, 2962, 2191, 1685, 1651, 1604, 1369, 1211; ^1^H NMR (250 MHz, DMSO-*d*_6_): 0.84 (s, 3H, CH_3_), 0.97 (s, 3H, CH_3_), 2.28 (d, 2H, *J* = 7.3 Hz, 2CH), 2.42 (d, 2H,* J* = 7.8 Hz, 2CH), 2.78 (s, 3H, SCH_3_), 3.83 (s, 3H, CH_3_), 4.32 (s, 1 H, CH), 5.33 (s, 2 H, NH_2_), 6.75–7.03 (d, 2H,* J* = 8.3 Hz ArH), 7.27–7.38 (d, 2H, *J* = 8.6 Hz, ArH), 7.64–7.75 (m, 4H, ArH); ^13^C NMR (62.9 MHz, DMSO-*d*_6_) δ: 27.33, 31.72, 76.26, 85.62, 94.22, 104.06, 116.41, 119.46, 123.54, 126.81, 131.54, 133.93, 135.22, 138.51, 141.62, 143.01, 148.32, 157.24, 164.87, 175.44, 176.86, 182.71, 185.31, 193.11, 193.82, 195.71; Anal. Calcd. For: C_26_H_27_N_3_O_2_S: C 70.09, H 6.11, N 9.43. O 7.18, S, 7.20. Found: C 70.06, H 6.16, N 9.48 O 7.12, S, 7.24; MS (EI) (m/z): 455.18 (M^+^).

## Results and discussion

**Figure 2:**
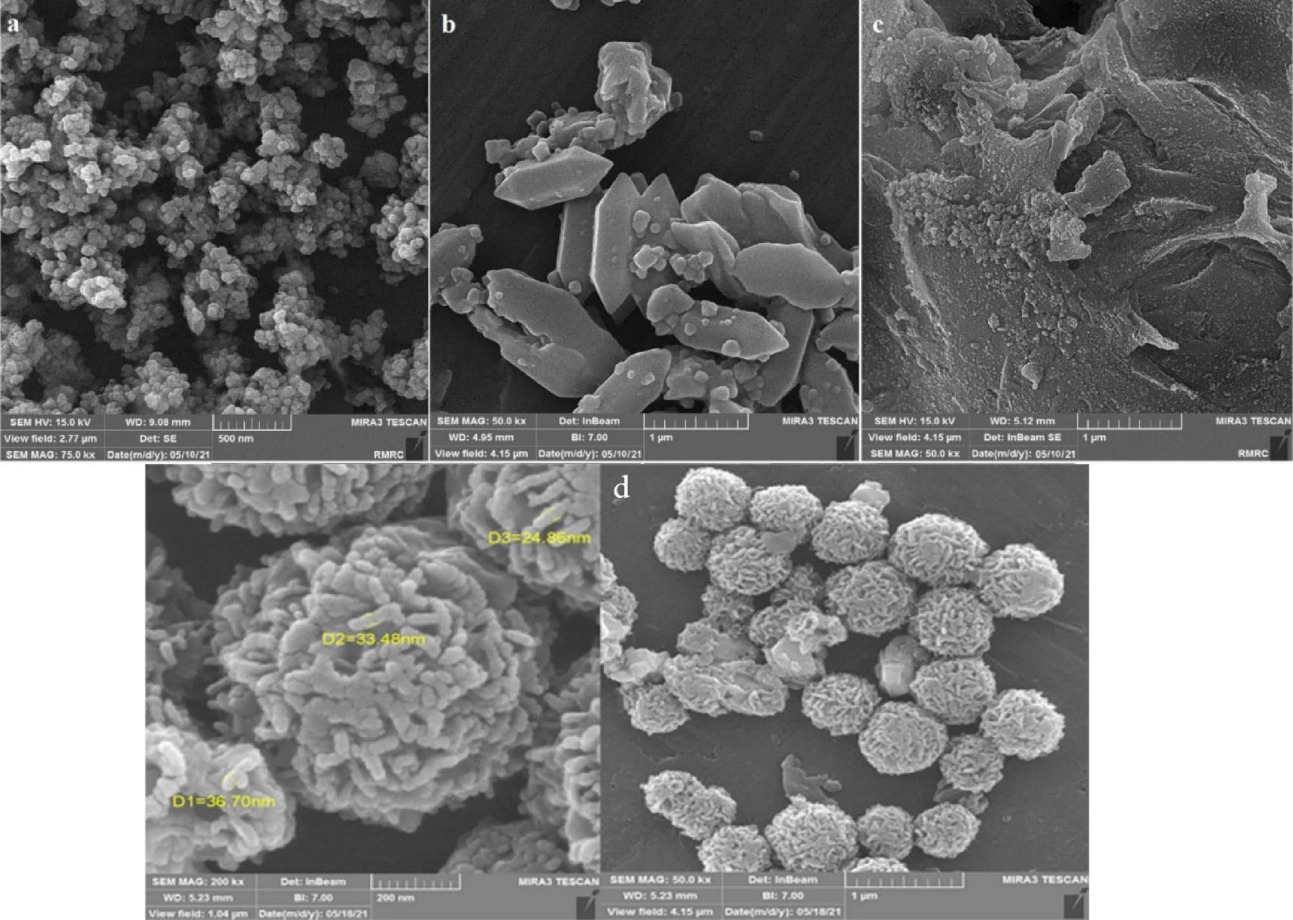
SEM analysis of (**a**) CuFe_2_O_4_, (**b**) IRMOF-3, (**c**) GO, and (**d**) IRMOF-3/GO/CuFe_2_O_4_.

**Figure 3:**
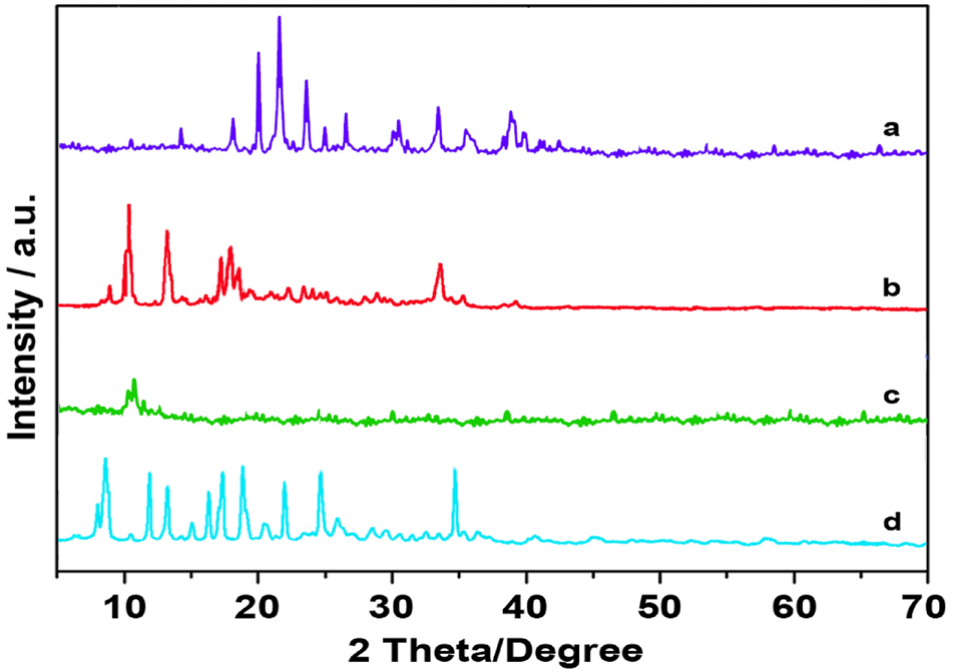
XRD patterns of (**a**) pure CuFe_2_O_4_, (**b**) IRMOF-3, (**c**) GO, and (**d**) IRMOF-3/GO/CuFe_2_O_4_.

**Figure 4:**
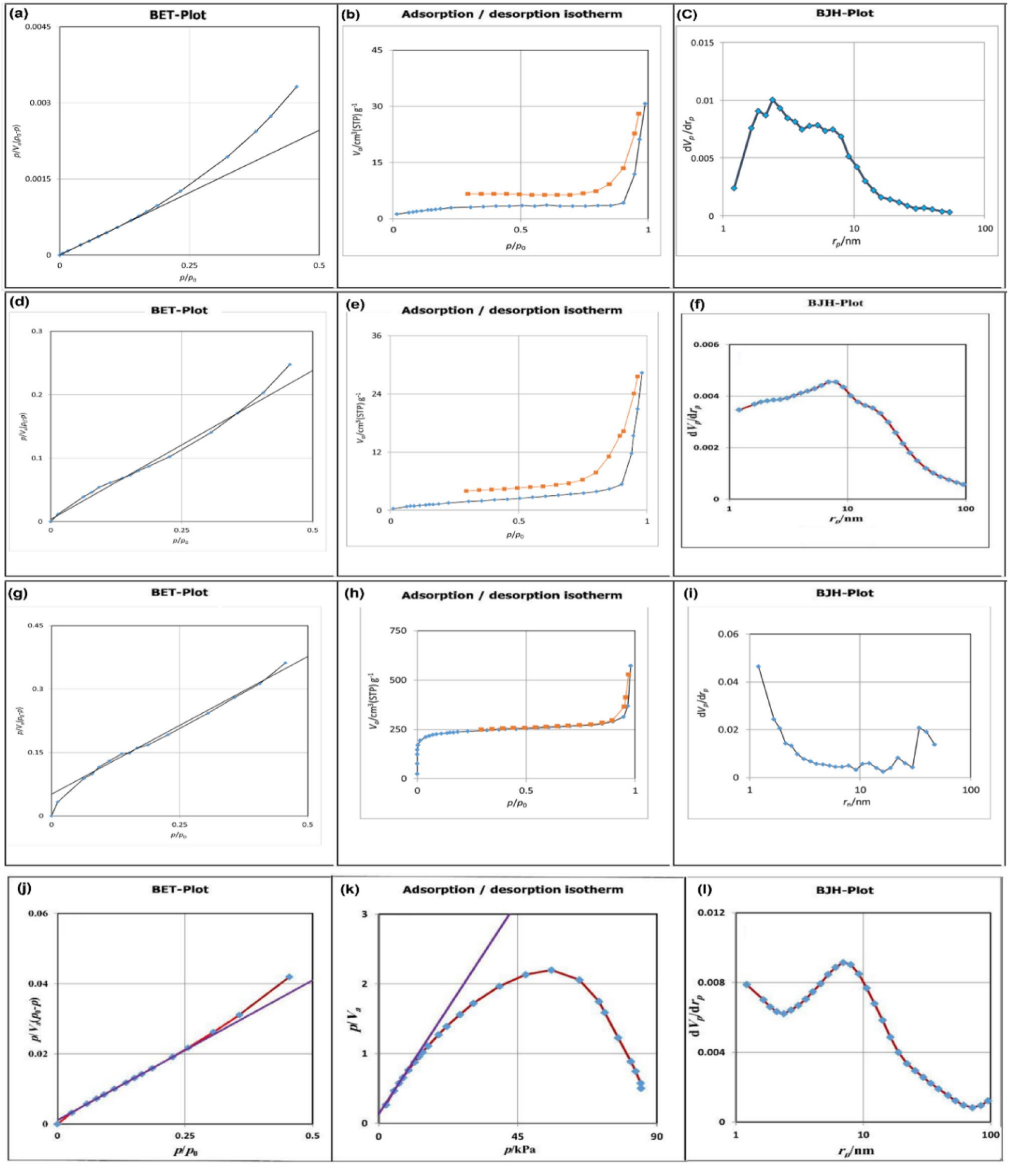
BET-plot of CuFe_2_O_4_ (**a**), adsorption/desorption of CuFe_2_O_4_ (**b**), BJH-plot of CuFe_2_O_4_ (**c**); BET-plot of GO (**d**), adsorption/desorption of GO (**e**), BJH-plot of GO (**f**); BET-plot of IRMOF-3 (**g**), adsorption/desorption of IRMOF-3 (**h**), BJH-plot of IRMOF-3 (**i**), BET-plot of IRMOF-3/GO/CuFe_2_O_4_ (**j**), adsorption/desorption of IRMOF-3/GO/CuFe_2_O_4_ (**k**), BJH-plot of IRMOF-3/GO/CuFe_2_O_4_ (**l**).

**Figure 5:**
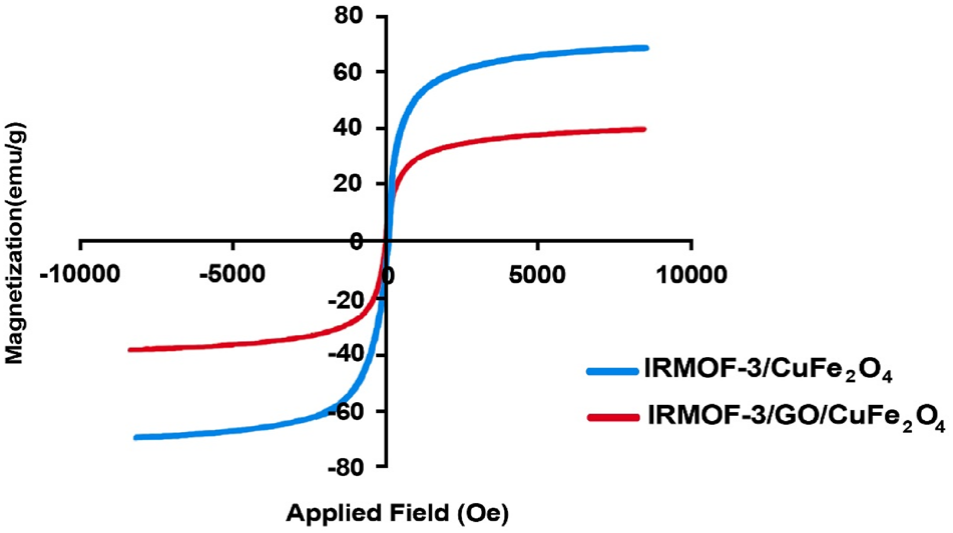
Magnetization curves of IRMOF-3/CuFe_2_O_4_ and IRMOF-3/GO/CuFe_2_O_4_.

IRMOF-3/GO/CuFe_2_O_4_ was successfully synthesized as mentioned in our previous study^31^. The structure of this catalyst was determined using EDX/Mapping, IR, and TGA analysis (See Supplementary Information, Figs. S2–S5)^31^, and also with other spectral techniques including SEM (Fig. 2), XRD (Fig. 3), BET (Fig. 4), and VSM (Fig. 5).

The microscopic morphology of the products was visualized by scanning electron microscopy (SEM). This analysis showed that CuFe_2_O_4_ is composed of relatively uniform quasispherical particles (Fig. 2a)^32^. SEM images of IRMOF-3 showed that this structure has a crystalline and rod shape, per previous reports (Fig. 2b)^33^. SEM image of the GO shows the particles of GO look very dense with the layers stacked together due to dispersive forces and strong specific interactions between the surface groups on the graphene-like layers (Fig. 2c)^34^. SEM images for the IRMOF-3/GO/CuFe_2_O_4_ nanocomposite are shown in Fig. 3d. As can be seen, the particles have regular and spherical shapes (Fig. 2d).

XRD pattern of the CuFe_2_O_4_ nanoparticles is shown in Fig. 3a. The peaks at 29.80°, 32.21°, 35.71°, 43.31°, 48.40°, 57.25°, 59.66° and 63.62° (2θ°) indicate the formation of the CuFe_2_O_4_ nanoparticles^35^. Also the peaks at 8.4°, 9.6°, 13.7°, 15.35°,19.39° and 24.62° (2θ°) show the presence of the IRMOF-3 frameworks (Fig. 3b)^36^. Here, the peak at 2θ = 11.1° indicates the creation of the GO (Fig. 3c)^37,38^. Fig. 2d presents the XRD pattern of IRMOF-3/GO/CuFe_2_O_4_. The peaks observed at 2θ = 29.80°, 32.21°, 35.71°, 43.31°, 48.40°, 57.25°, 59.66° and 63.62° are related to the CuFe_2_O_4_. Also, the peaks at 2θ of 8.4°, 9.6°, 13.7°, 15.35°, 19.39°, and 24.62° show the presence of the IRMOF-3 frameworks, while the peak at 2θ = 11.1° indicates the creation of the GO, confirming the formation of the nanocomposite. These results demonstrate that the crystalline structure of the CuFe_2_O_4_ and IRMOF-3 materials was unchanged and remained intact during the catalyst preparation.

Nitrogen adsorption/desorption isotherms were used to study the specific surface area and pore volume distribution of nanostructures including CuFe_2_O_4_, GO, IRMOF-3, and IRMOF-3/GO/CuFe_2_O_4_ by the Brunauer–Emmett–Teller (BET) approach (Fig. 4). The CuFe_2_O_4_ represented a BET surface area, total pore volume, and the average pore diameter of 34.65 m^2^ g^−1^, 0.138 cm^3^ g^−1^, and 1.17 nm respectively. In comparison, these analyses for GO, IRMOF-3 and IRMOF-3/GO/CuFe_2_O_4_ gave values of 48.51 m^2^ g^−1^, 0.182 cm^3^ g^−1^, and 1.24 nm, 884.82 m^2^ g^−1^, 0.887 cm^3^ g^−1^, and 1.42 nm, and 456.29 m^2^ g^−1^, 0.435 cm^3^ g^−1^ and, 1.65 nm, respectively (Fig. 4 and Table 1). The adsorption–desorption isotherm of all the four samples exhibits a reversible type-II adsorption isotherm, indicating the presence of micro-and macro-pores^39^. The decreasing BET surface area and total pore volume can be attributed to incorporating of IRMOF-3/CuFe_2_O_4_ groups inside the pure GO. However, the open cavities and high surface areas were retained, which benefited the free dispersal of the reactant and product.

**Table 1:** Measured BET surface areas and pore volumes of CuFe_2_O_4_, GO, IRMOF-3 and IRMOF-3/GO/CuFe_2_O_4_.

Sample	BET Surface area (m^2^/g)	Pore volume (cm^3^/g)	Pore size (nm)
CuFe_2_O_4_	34.65	0.138	1.17
GO	48.51	0.182	1.24
IRMOF-3	884.82	0.887	1.42
IRMOF-3/GO/CuFe_2_O_4_	456.29	0.435	1.65

The so-called VSM analysis was further conducted to investigate the magnetic behavior of the IRMOF-3/CuFe_2_O_4_, and IRMOF-3/GO/CuFe_2_O_4_ nanocomposite, with the outcomes presented in Fig. 5. According to the results, the value of magnetic saturation was measured at 67.45, and 38.45 emu/g for the IRMOF-3/CuFe_2_O_4_ and IRMOF-3/GO/CuFe_2_O_4_ nanocomposite, respectively^40^.

The performance of the prepared IRMOF-3/GO/CuFe_2_O_4_ nanocomposite was evaluated using the catalyst in the preparation of 2-amino-4-aryl-6-substituted pyridine-3,5-dicarbonitrile and hydroquinoline-3-carbonitrile derivatives. To this end, a three-component reaction of 4-bromobenzaldehyde, *p*-toluidine, and malononitrile was chosen as a model study for synthesizing 2-amino-4-(4-bromophenyl)-6-(*p*-tolylamino)pyridine-3,5-dicarbonitrile (4e). In addition, the reaction of *p*-toluidine, 4-bromobenzaldehyde, dimedone, and malononitrile was considered a model reaction for the preparation of 2-amino-4-(4-bromophenyl)-7,7-dimethyl-5-oxo-1-(*p*-tolyl)-1,4,5,6,7,8-hexahydroquinoline-3-carbonitrile (6b) (Table 2). The model reactions were investigated by different conditions, including catalyst, solvent, and amount of catalyst.

**Table 2:** The influence of various catalysts and solvents in the preparation of 4e and 6b compounds.


Entry	Compound	Solvent	Catalyst	T (°C)	Time (min)	Yield%
1	4e	C_2_H_5_OH	Fe_3_O_4_ NPs	u.s	25	68
2	4e	C_2_H_5_OH	CuFe_2_O_4_ NPs	u.s	25	70
3	4e	C_2_H_5_OH	MgO NPs	u.s	35	75
4	4e	C_2_H_5_OH	SiO_2_ NPs	u.s	45	65
5	4e	C_2_H_5_OH	IRMOF-3/CuFe_2_O_4_	u.s	25	85
6	4e	C_2_H_5_OH	CuI NPs	u.s	30	75
7	4e	C_2_H_5_OH	GO NPs	u.s	35	80
**8**	**4e**	**C**_**2**_**H**_**5**_**OH**	**IRMOF-3/GO/CuFe**_**2**_**O**_**4**_	**u.s**	**15**	**92**
9	6b	C_2_H_5_OH	Fe_3_O_4_ NPs	u.s	35	80
10	6b	C_2_H_5_OH	CuFe_2_O_4_ NPs	u.s	40	82
11	6b	C_2_H_5_OH	MgO NPs	u.s	60	55
12	6b	C_2_H_5_OH	SiO_2_ NPs	u.s	45	65
13	6b	C_2_H_5_OH	IRMOF-3/CuFe_2_O_4_	u.s	30	72
14	6b	C_2_H_5_OH	CuI NPs	u.s	35	75
15	6b	C_2_H_5_OH	GO NPs	u.s	40	78
**16**	**6b**	**C**_**2**_**H**_**5**_**OH**	**IRMOF-3/GO/CuFe**_**2**_**O**_**4**_	**u.s**	**25**	**90**
17	4e	H_2_O	IRMOF-3/GO/CuFe_2_O_4_	u.s	35	75
18	4e	CHCl_3_	IRMOF-3/GO/CuFe_2_O_4_	u.s	45	45
19	4e	DMF	IRMOF-3/GO/CuFe_2_O_4_	u.s	35	75
**20**	**4e**	**C**_**2**_**H**_**5**_**OH**	**IRMOF-3/GO/CuFe**_**2**_**O**_**4**_	**u.s**	**15**	**92**
21	4e	PhCH_3_	IRMOF-3/GO/CuFe_2_O_4_	u.s	45	40
22	4e	–	IRMOF-3/GO/CuFe_2_O_4_	u.s	45	–-
23	6b	H_2_O	IRMOF-3/GO/CuFe_2_O_4_	u.s	60	65
24	6b	CHCl_3_	IRMOF-3/GO/CuFe_2_O_4_	u.s	60	50
25	6b	DMF	IRMOF-3/GO/CuFe_2_O_4_	u.s	45	65
**26**	**6b**	**C**_**2**_**H**_**5**_**OH**	**IRMOF-3/GO/CuFe**_**2**_**O**_**4**_	**u.s**	**25**	**90**
27	6b	PhCH_3_	IRMOF-3/GO/CuFe_2_O_4_	u.s	60	35
28	6b	–	IRMOF-3/GO/CuFe_2_O_4_	u.s	60	–-

Significant values are in bold.

Firstly, the preparation of compounds 4e and 6p was considered in the occurrence of different catalysts like Fe_3_O_4_, CuFe_2_O_4_, MgO, SiO_2_, IRMOF-3/CuFe_2_O_4_, CuI, GO, and IRMOF-3/GO/CuFe_2_O_4_ nanostructures under ultrasound irradiations in EtOH as solvent (0.01 g of each catalyst was used). The best results were obtained (Table 2), when using IRMOF-3/GO/CuFe_2_O_4_ nanocomposite as catalyst (Table 2, entries 8 and 16). Afterward, it was tried to investigate the influence of various solvents like water, chloroform, dimethylformamide, ethanol, toluene, and solvent-free conditions in synthesizing of 2-amino-4-(4-bromophenyl)-6-(*p*-tolylamino)pyridine-3,5-dicarbonitrile (4e) in the presence of IRMOF-3/GO/CuFe_2_O_4_ (0.01 g) (Table 2, entries 17–22). Table 2 shows no product was achieved without any solvent under ultrasonic irradiation. Furthermore, it was concluded that the presence of ethanol as solvent provided the best outcome considering reaction time and yield of the corresponding product. Subsequently, the preparation of hexahydroquinoline-3-carbonitrile (6b) was also examined using different polar and nonpolar solvents (Table 2, entries 23–28). As indicated in this Table 2, using EtOH as solvent provided the best reaction conditions (Table 2, entries 20 and 26).

In the next stage, it was decided to assess the optimum amount of the nanocatalyst for preparing of 4e and 6b. Different amounts of the catalyst were used for preparing 2-amino-4-(4-bromophenyl)-6-(*p*-tolylamino)pyridine-3,5-dicarbonitrile and 2-amino-4-(4-bromophenyl)-7,7-dimethyl-5-oxo-1-(p-tolyl)-1,4,5,6,7,8-hexahydroquinoline-3-carbonitrile (Table 3). The model was run using various amounts of the catalyst from 0.001 to 0.007 g. Eventually, the optimal amounts were found to be 0.003 and 0.005 g for compounds 4e and 6p, respectively.

**Table 3:** The effect of different quantities of the catalyst in model studies.

Entry	Compound	Catalyst (g)	Time (min)	Yield %
1	4e	0.001	30	70
2	4e	0.002	25	85
**3**	**4e**	**0.003**	**15**	**92**
4	4e	0.005	15	92
5	6b	0.001	60	55
6	6b	0.002	45	75
**7**	6b	0.003	40	85
**8**	**6b**	**0.005**	**25**	**90**
7	6b	0.007	25	88

Significant values are in bold.

Some aromatic aldehydes and amines were used to investigate the catalytic efficiency and performance of the IRMOF-3/GO/CuFe_2_O_4_ on synthesizing various pyridine and hydroquinoline derivatives under optimized reaction conditions. After conducting some experiments, we obtained some 2-amino-4-aryl-6-substituted-pyridine-3,5-dicarbonitriles and hydroquinolone-3-carbonitriles in good to excellent yields within short reaction times. As illustrated in Table 4, different aldehydes and anilines with various substituents could participate in the multi-component synthesis of corresponding heterocyclic compounds. However, as indicated, benzaldehydes with electron-withdrawing groups reacted faster than electron-donating ones. Moreover, the aromatic amines having electron-releasing groups reacted faster than anilines containing electron-withdrawing groups. According to the mechanism pathway, the first step of the mechanism is the Knoevenagel condensation between malononitrile and aromatic aldehyde. Obliviously, the presence of electron-withdrawing groups on the benzaldehydes leads to a faster reaction due to more electrophilicity of the carbon group of the carbonyl^41,42^. While using IRMOF-3/GO/CuFe_2_O_4_ as a catalyst serves as a Lewis acid catalyst and increases the electrophilicity of the carbonyl groups of aldehydes. The synergic effects of both electron-withdrawing groups and Lewis acidity of IRMOF-3/GO/CuFe_2_O_4_ increase the reaction rate. Nevertheless, against the electron-withdrawing groups, the existence of electron-donating groups lowers the speed of the reaction, because of a slower nucleophilic attack on carbonyl groups of aldehydes.

**Table 4:** Preparation of 1,8-dioxo-decahydro-acridines (4a–r) and 1,8-dioxo-octahydro-xanthenes (6a–d) using IRMOF-3/GO/CuFe_2_O_4_ under ultrasound irradiations.

Entry	Ar	Ar'	Product	Time (min)	Yield (%)^a^
1	C_6_H_5_	C_6_H_5_	4a	20	88
2	*p*-MeC_6_H_4_	C_6_H_5_	4b	15	90
3	*p*-MeC_6_H_4_	*p*-ClC_6_H_4_	4c	15	95
4	*p*-MeC_6_H_4_	*p*-OMeC_6_H_4_	4d	25	85
5	*p*-MeC_6_H_4_	*p*-BrC_6_H_4_	4e	15	92
6	*p*-OMeC_6_H_4_	C_6_H_5_	4f	15	90
7	*p*-OMeC_6_H_4_	*p*-BrC_6_H_4_	4g	15	95
8	*p*-OMeC_6_H_4_	*p*-OMeC_6_H_4_	4h	20	90
9	*p*-OMeC_6_H_4_	*p*-ClC_6_H_4_	4i	15	97
10	*p*-OMeC_6_H_4_	*p*-CNC_6_H_4_	4j	15	95
11	*p*-OMeC_6_H_4_	*p*-SMeC_6_H_4_	4k	25	88
12	*p*-ClC_6_H_4_	C_6_H_5_	4l	20	85
13	*p*-ClC_6_H_4_	*p*-BrC_6_H_4_	4m	15	92
14	*p*-ClC_6_H_4_	*p*-OMeC_6_H_4_	4n	25	85
14	C_6_H_5_	C_6_H_5_	4n	30	90
15	*p*-MeC_6_H_4_	*p*-MeC_6_H_4_	4o	35	88
16	*p*-MeC_6_H_4_	*p*-BrC_6_H_4_	4p	25	90
17	*p*-MeC_6_H_4_	*p*-ClC_6_H_4_	4q	25	95
18	*p*-MeC_6_H_4_	*p*-OMeC_6_H_4_	4r	40	85
19	*p*-MeC_6_H_4_	*p*-NO_2_C_6_H_4_	6a	25	95
20	*p*-MeC_6_H_4_	*p-BrC*_*6*_*H*_*4*_	6b	15	90
21	*p*-OMeC_6_H_4_	*p*-CNC_6_H_4_	6c	30	85
22	*p*-OMeC_6_H_4_	*p*-SMeC_6_H_4_	6d	35	90

^a^Isolated yield.

The catalyst efficiency and usability for synthesizing 2-amino-4-aryl-6-substituted pyridine-3,5-dicarbonitrile and hydroquinoline-3-carbonitrile derivatives were compared with those of some previously reported catalysts. As presented in Table 5, IRMOF-3/GO/CuFe_2_O_4_ is better than previously reported catalysts in saving time, and energy, and excellent yields of the products (Table 5).

**Table 5:** Comparison of the outcomes of the production of 2-amino-4-aryl-6-substituted pyridine-3,5-dicarbonitrile and hydroquinoline-3-carbonitrile derivatives by means of different catalysts.

	Entry	Catalyst	Conditions	Time/yield (%)	References
	1	ZnCl_2_	EtOH/Stir.80 °C	5 h /67–88	^43^
	2	DMAPL	MeOH/r.t	4 h/62–86	^44^
	3	ZnCl_2_/AlCl_3_/FeCl_3_	EtOH/Reflux	6 h/73–95	^41^
	4	KOH	EtOH/r.t	2 h/77–91	^42^
	**5**	**IRMOF-3/GO/CuFe**_**2**_**O**_**4**_	**EtOH/ultrasound**	**15 min/ 85–97**	**This work**
	1	Piperidine	EtOH/Reflux	6 h /64–75	^45^
	2	[bmim^+^][BF_4_^−^]	Stir.(90 °C)	5 h/94–99	^46^
	3	MNP@BSAT@Cu(OAc)_2_	EtOH/r.t	25 min/80–94	^47^
	4	Zr(HPO_4_)_2_	Solvent-free/80 °C	2 h/65–98	^48^
	**5**	**IRMOF-3/GO/CuFe**_**2**_**O**_**4**_	**EtOH/ultrasound**	**15 min/85–95**	**This work**

Significant values are in bold.

### Reuse of catalyst

**Figure 6:**
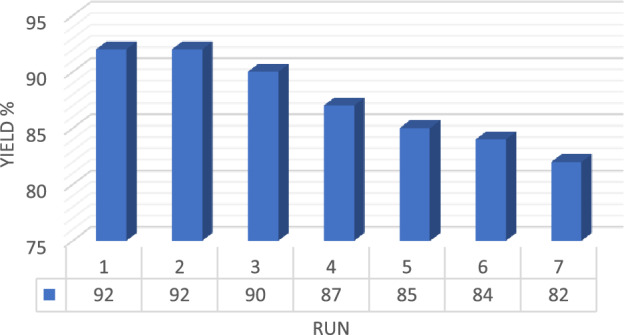
Reusability of the IRMOF-3/GO/CuFe_2_O_4_ catalyst.

**Figure 7:**
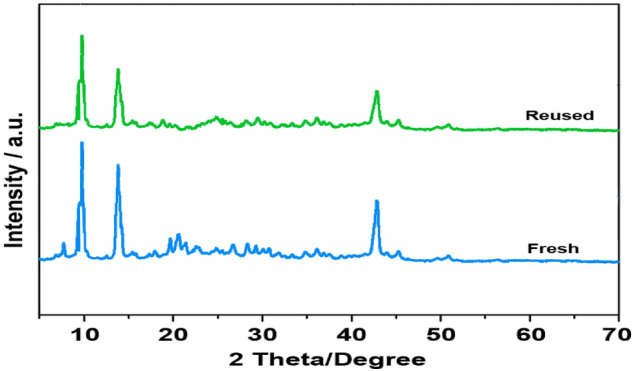
XRD pattern of the recovered IRMOF-3/GO/CuFe_2_O_4_.

In this research, the reusability of IRMOF-3/GO/CuFe_2_O_4_ composite was investigated for the three-component reaction of 4-bromobenzaldehyde, *p*-toluidine, and malononitrile (compound 4e). Upon completion of the reaction, the IRMOF-3/GO/CuFe_2_O_4_ nanostructure was separated using a centrifuge instrument and washed with dichloromethane. The obtained recovered catalyst was dried at 40 °C for 24 h. As indicated in Fig. 6, the recovered catalyst could be used 7 times. Furthermore, the XRD technique was used to demonstrate the efficiency and consistency of the rescue IRMOF-3/GO/CuFe_2_O_4_ after seven runs uses. As indicated, there is no considerable differences between X-ray diffraction of the fresh IRMOF-3/GO/CuFe_2_O_4_ and the recovered catalyst, confirming the good stability of the prepared nanocatalyst after 7 runs (Fig. 7).

**Figure 8:**
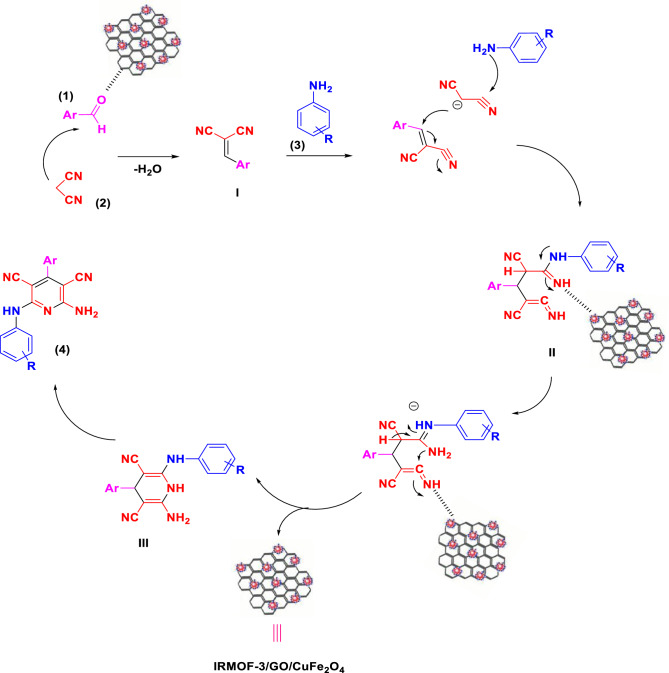
The proposed mechanism to synthesize pyridine derivatives using IRMOF-3/GO/CuFe_2_O_4_.

Figure 8 shows a reasonable mechanism for synthesizing pyridine derivatives (4a–4r) catalyzed by IRMOF-3/GO/CuFe_2_O_4_, This result is, supported by previous studies^41,42^. IRMOF-3/GO/CuFe_2_O_4_ was assumed to serve as Lewis acids that causes increasing electrophilicity of the carbonyl groups, double and triple bonds in substrates and intermediate via a strong coordination bond^17,49^. Initially, the mechanistic approach includes the Knoevenagel condensation of aromatic aldehyde and malononitrile, creating adduct **I**. Then, adding anilines to intermediate **I** was followed by involving malononitrile to make intermediate **II**. Tautomerization and intramolecular cyclization on intermediate **II** produced intermediate **III** with a dihydropyridine moiety. Finally, 2-amino-6-alkylamino-3,5-dicyanopyridines **4** was prepared by aromatization of intermediate **III**. Additionally, the catalytic behavior of IRMOF-3/GO/CuFe_2_O_4_ on the quinoline synthesis was identical to the described mechanism for synthesizing pyridine derivatives.

## Conclusion

The present work aimed to present a convenient and facile technique to prepare magnetic copper ferrite nanoparticles supported on IRMOF-3/GO. This magnetic heterogeneous and reusable nano catalyst to prepare heterocyclic compounds via the reactions between of various aromatic aldehydes, malononitrile, diverse primary amines and dimedone under ultrasound irradiations. The methods for characterizing the IRMOF-3/GO/CuFe_2_O_4_ were SEM, BET, FT-IR, XRD, TGA, and EDX. It was indicated that this catalyst is effective for synthesis of pyridines and quinoline derivatives. Moreover, this technique has good benefits like eco-friendly, higher performance and a very convenient working technique.

## Supplementary Information

Supplementary Information.

## Data Availability

All data generated or analyzed during this study are included in this published article [and its supplementary information file].
