# Supplementary material for: An efficient protocol for the synthesis of pyridines and hydroquinolones using IRMOF-3/GO/CuFe2O4 composite as a magnetically separable heterogeneous catalyst
Source: Sci Rep. 2023 Jun 5;13:9089. doi: 10.1038/s41598-023-36115-2 (PMC10241797; doi:10.1038/s41598-023-36115-2)
Supplement: Supplementary file 1 — Supplementary Information. [file 41598_2023_36115_MOESM1_ESM.docx]

***Supporting Information***

**An Efficient Protocol for the Synthesis of Pyridines and Hydroquinolones Using IRMOF-3/GO/CuFe_2_O_4_ Composite as a Magnetically Separable Heterogeneous Catalyst**

Mohammad Ali Ghasemzadeh^a^*****, Boshra Mirhosseini-Eshkevari^a^, Jaber

Dadashi^b^

*^a^ Department of Chemistry, Qom Branch, Islamic Azad University, Qom, I. R. Iran*

*Post Box: 37491-13191, I. R. Iran.*

*^b^ Catalysts and Organic Synthesis Research Laboratory, Department of Chemistry, Iran University of Science and Technology, Tehran, 16846-13114, Iran*

*Corresponding author, E-mail address: m.a.qasemzade@gmail.com;qasemzade.a@gmail.com*

**Fig. S1.** Preparation of IRMOF-3/GO/CuFe_2_O_4_.


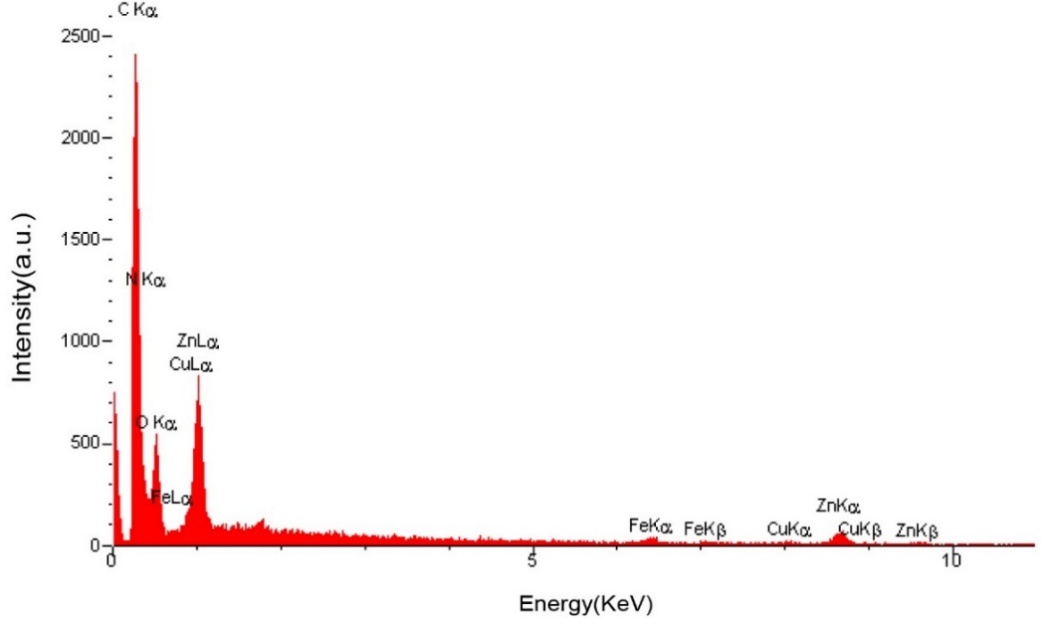


**Fig. S2.** The EDX spectrum of IRMOF-3/GO/CuFe_2_O_4_.


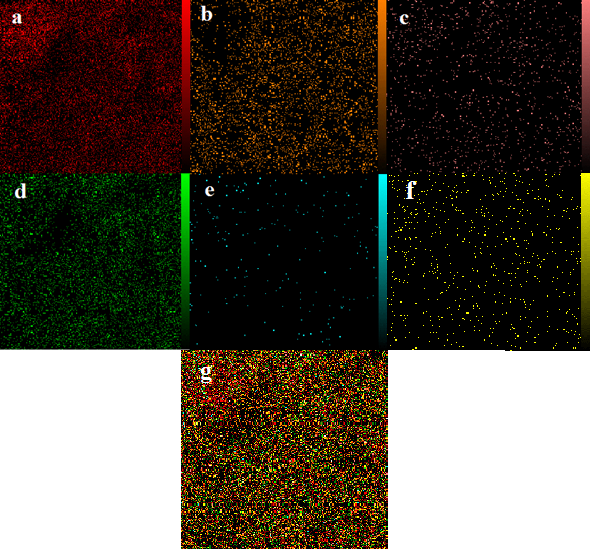


**Fig. S3.** EDX-Mapping of the (a) C, (b) Fe, (c) N, (d) O, (e) Cu, (f) Zn, and (g) IRMOF-3/GO/CuFe_2_O_4_.


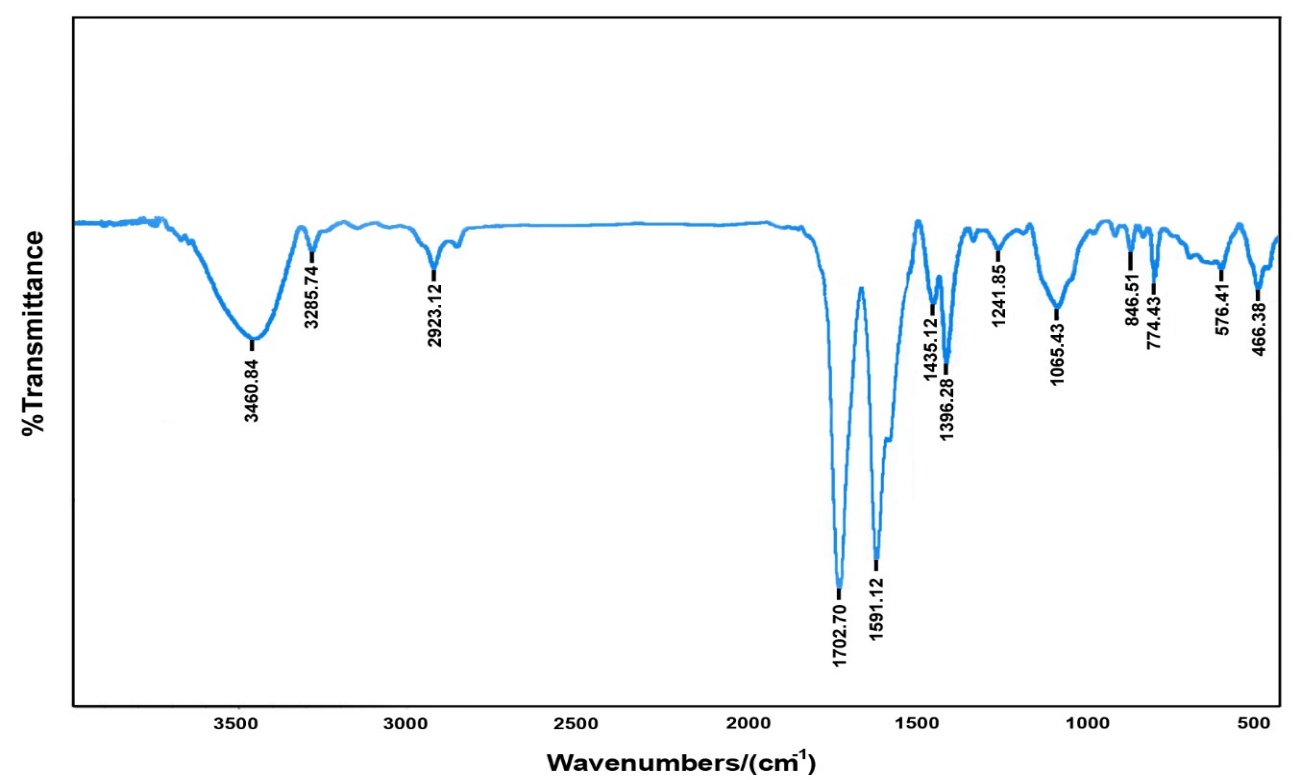


**Fig. S4.** FT-IR spectrum of IRMOF-3/GO/CuFe_2_O_4_.


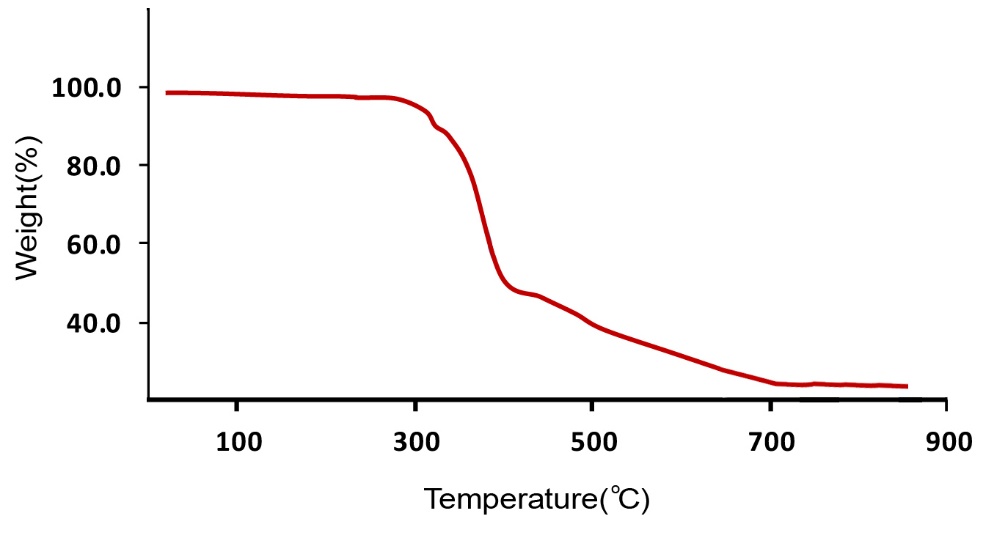


**Fig. S5.** TGA curve of IRMOF-3/GO/CuFe_2_O_4_.

**2-amino-4-(4-cyanophenyl)-6-((4-methoxyphenyl) amino) pyridine-3,5-dicarbonitrile 4j.** Yellow solid; m.p. 223-225°C. IR spectrum ν, cm^–1^: 3365, 3172, 3051, 2932, 2370, 1705,1674, 1591, 1458, 1205; ^1^H NMR (250 MHz, DMSO-*d*_6_) : 3.84 (s, 3H, CH_3_), 5.43 (s, 2 H, NH_2_), 6.76-7.04 (d, 2H, J = 8.2 Hz, ArH), 7.28-7.36 (d, 2H, J = 8.3 Hz, ArH), 7.65-7.77 (m, 4H, ArH), 9.85 (s, 1H, NH); ^13^C NMR (62.9 MHz, DMSO-*d*_6_) δ: 27.33, 31.39, 68.62, 76.82, 87.26, 101.03, 112.53, 123.45, 131.34, 133.43, 134.25, 138.56, 144.43, 146.33, 148.62, 156.47, 165.38, 168.28, 170.21, 183.31, 188.22; Anal. Calcd. For: C_21_H_14_N_6_O: C 68.84, H 3.85, N 22.94. O 4.37. Found: C 76.88, H 3.82, N 22.92 O 4.35; MS (EI) (m/z): 366.12 (M^+^).


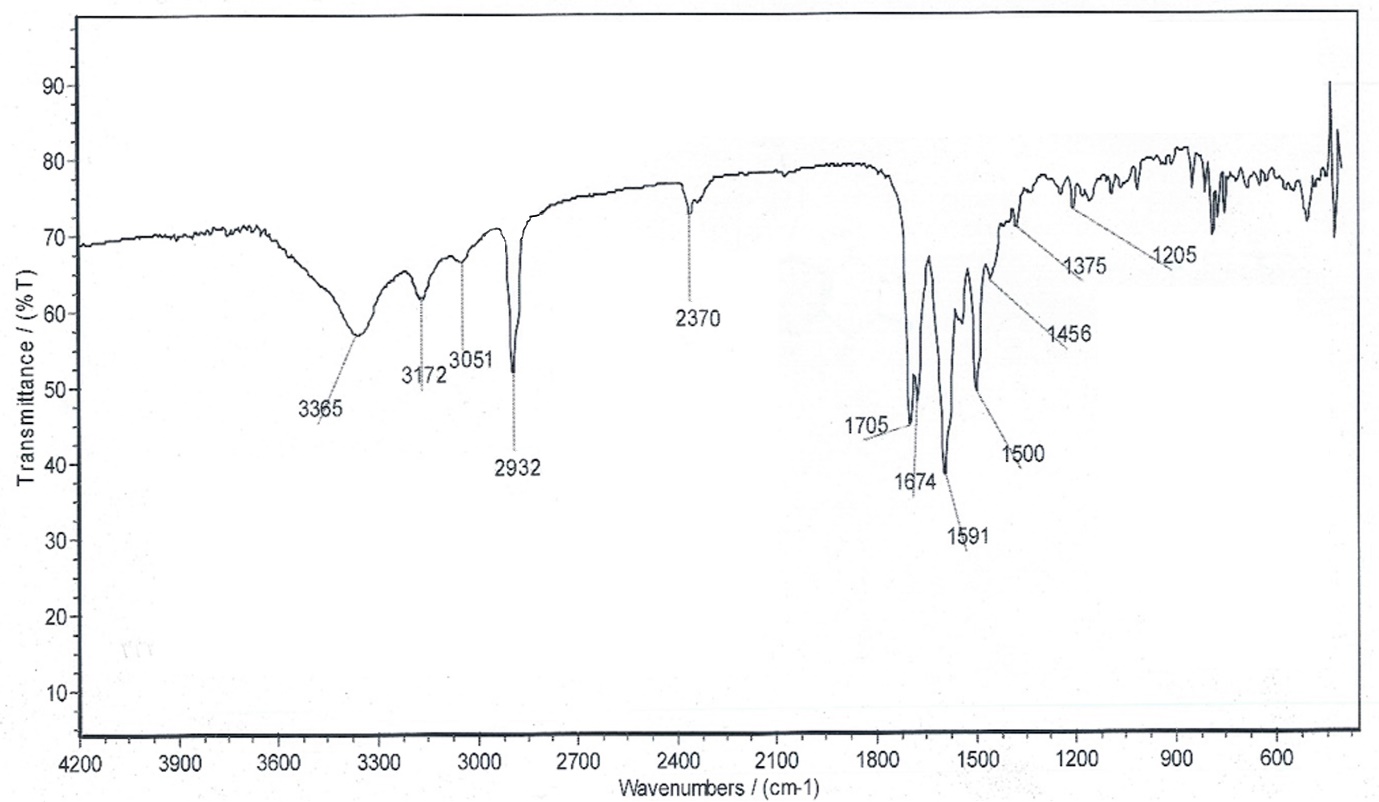


**Fig. S6.** FT-IR spectrum of 4j.


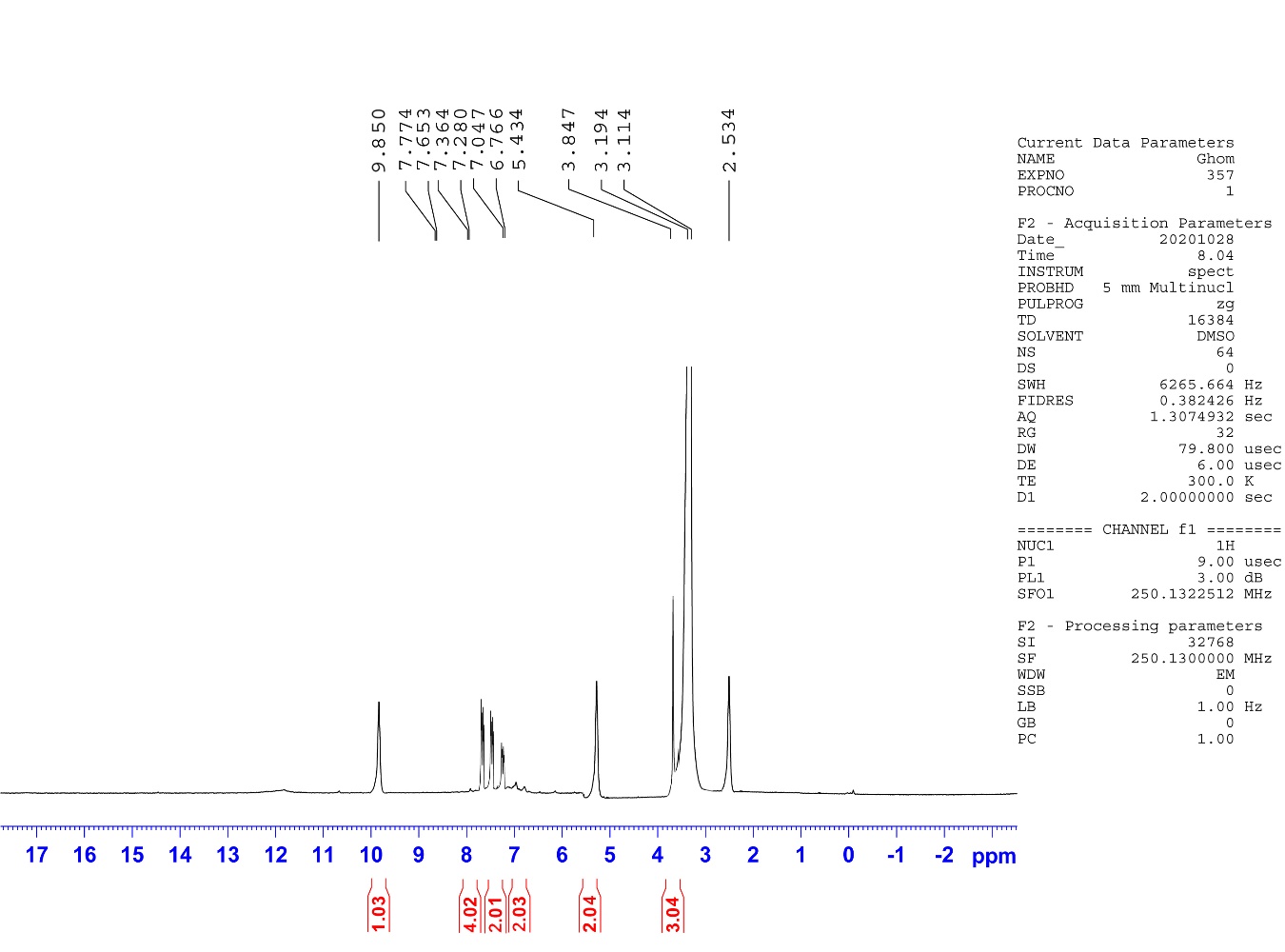


**Fig. S7.** ^1^H-NMR spectrum of 4j.


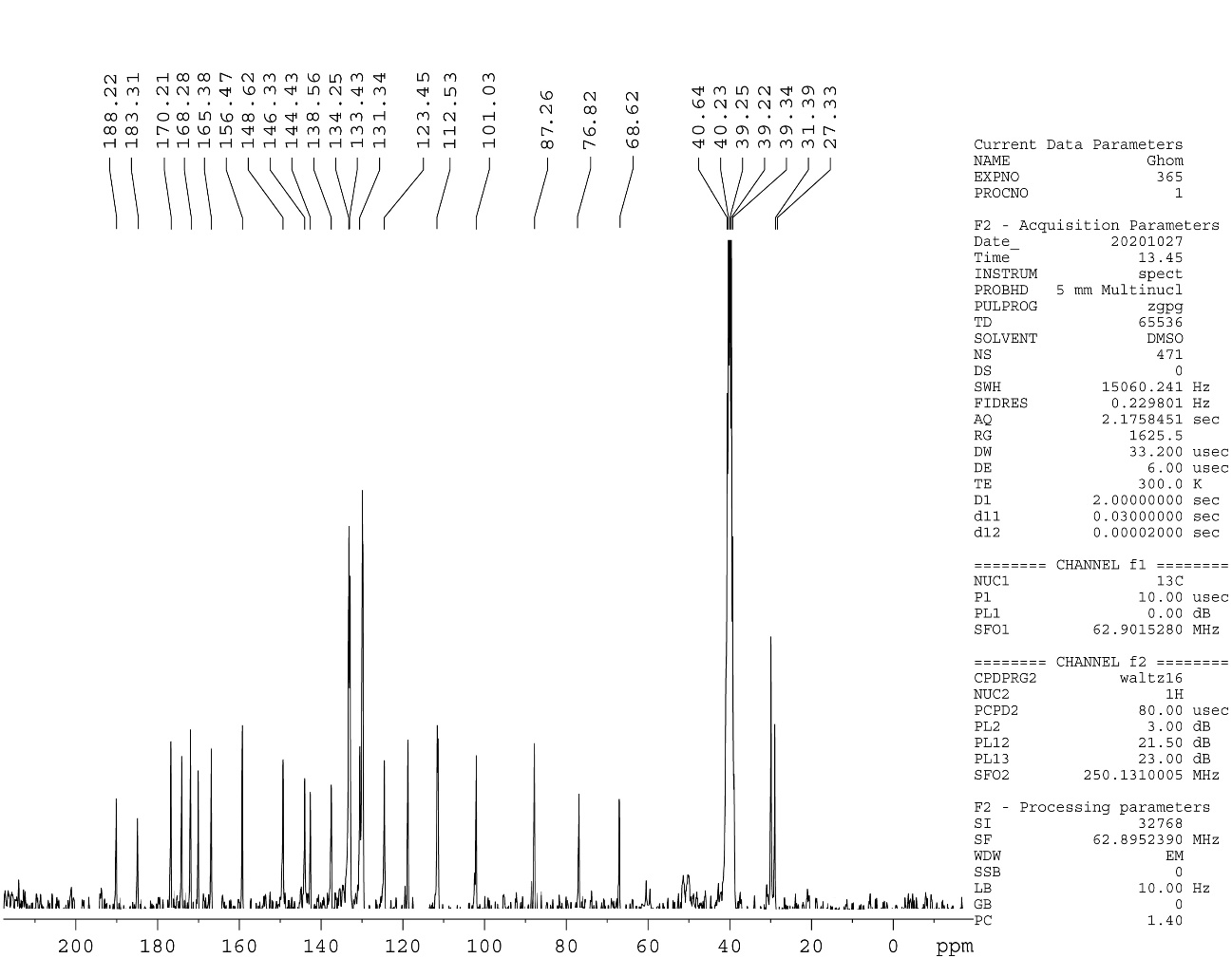


**Fig. S8.** ^13^C-NMR spectrum of 4j.

**2-amino-6-((4-methoxyphenyl)amino)-4-(4-(methylthio)phenyl)pyridine-3,5 dicarbonitrile 4k.** Yellow solid; m.p. 210-212°C. IR spectrum ν, cm^–1^: 3363, 3170, 2555, 21381, 1701, 1678, 1593, 1458, 1377, 1207; ^1^H NMR (250 MHz, DMSO-*d*_6_) : 2.36 (s, 3H, CH_3_), 3.72 (s, 3H, CH_3_), 5.48 (s, 2 H, NH_2_), 6.76-7.04 (d, 2H, J = 8.2 Hz, ArH), 7.27-7.36 (d, 2H, J = 8.3 Hz, ArH), 7.65-7.78 (m, 4H, ArH), 9.82 (s, 1H, NH); ^13^C NMR (62.9 MHz, DMSO-*d*_6_) δ: 28.39, 32.33, 67.62, 77.82, 86.26, 102.03, 113.52, 125.44, 132.34, 134.43, 136.26, 139.56, 146.44, 148.33, 149.69, 154.47, 162.34, 164.28, 171.21, 181.32, 187.21; Anal. Calcd. For: C_21_H_17_N_5_OS: C 65.10, H 4.42, N 18.08. O 4.13, S 8.27. Found: C 65.14, H 4.45, N 18.04, O 4.316, S 8.25.; MS (EI) (m/z): 387.12 (M^+^).


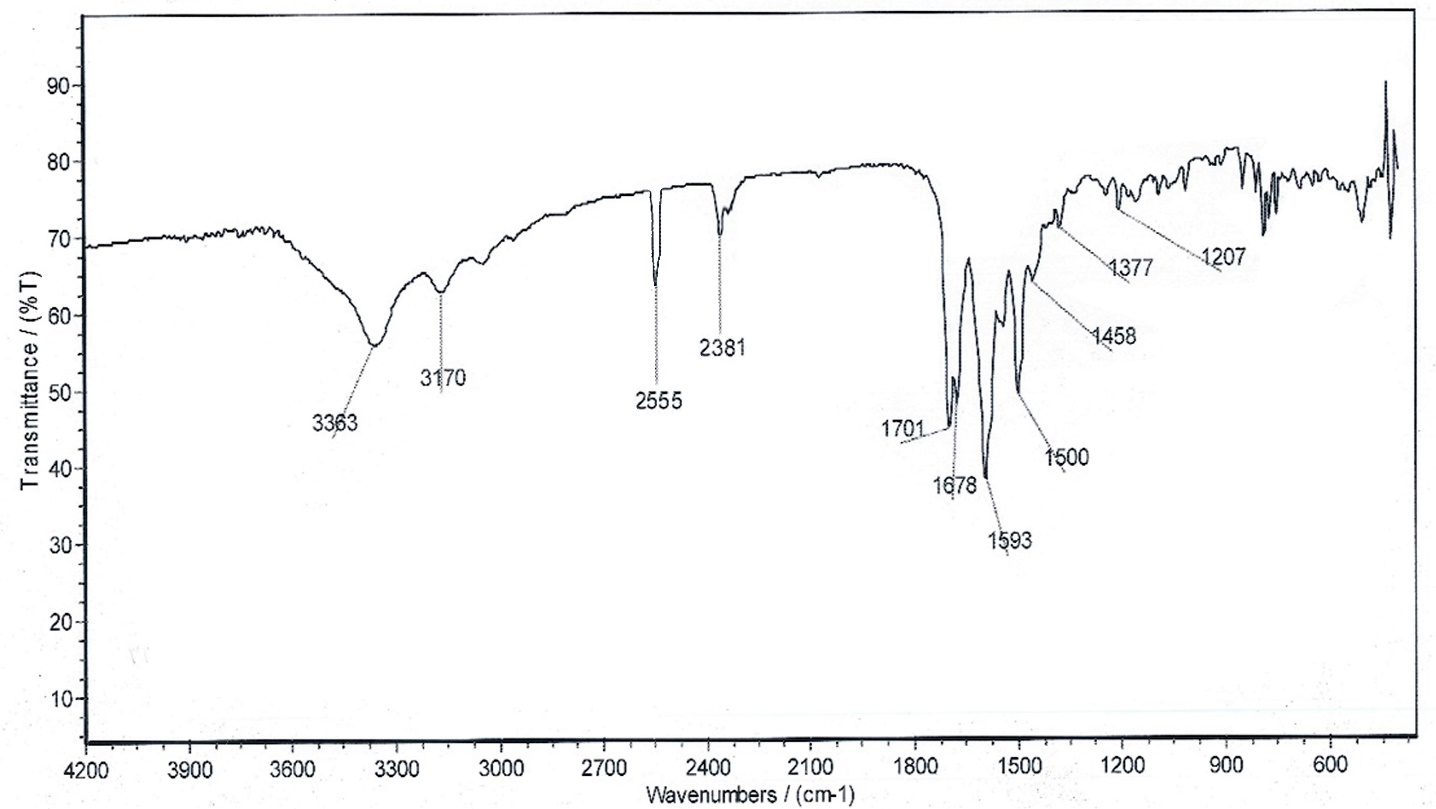


**Fig. S9.** FT-IR spectrum of **4k**.


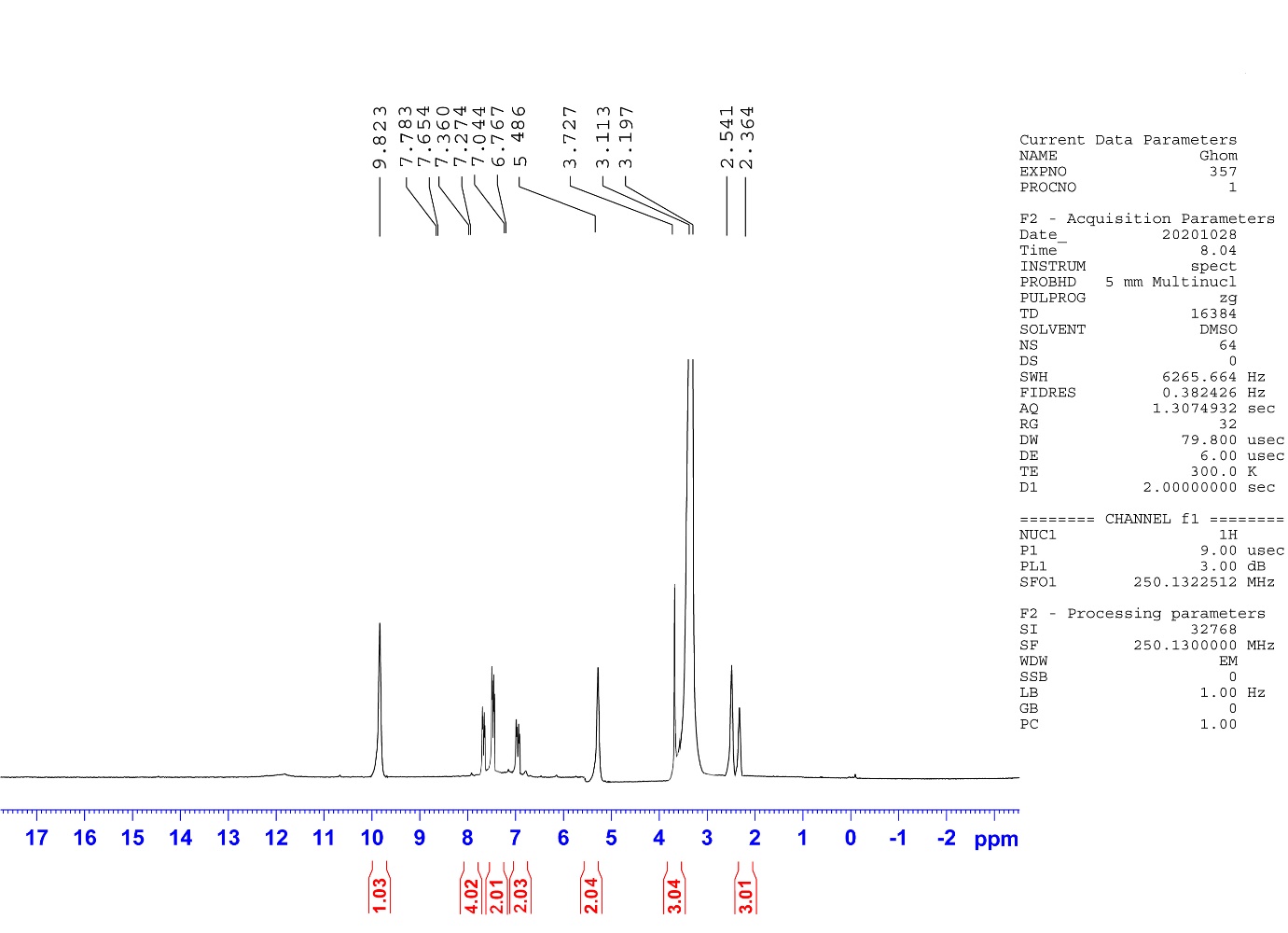


**Fig. S10.** ^1^H-NMR spectrum of 4k.

**
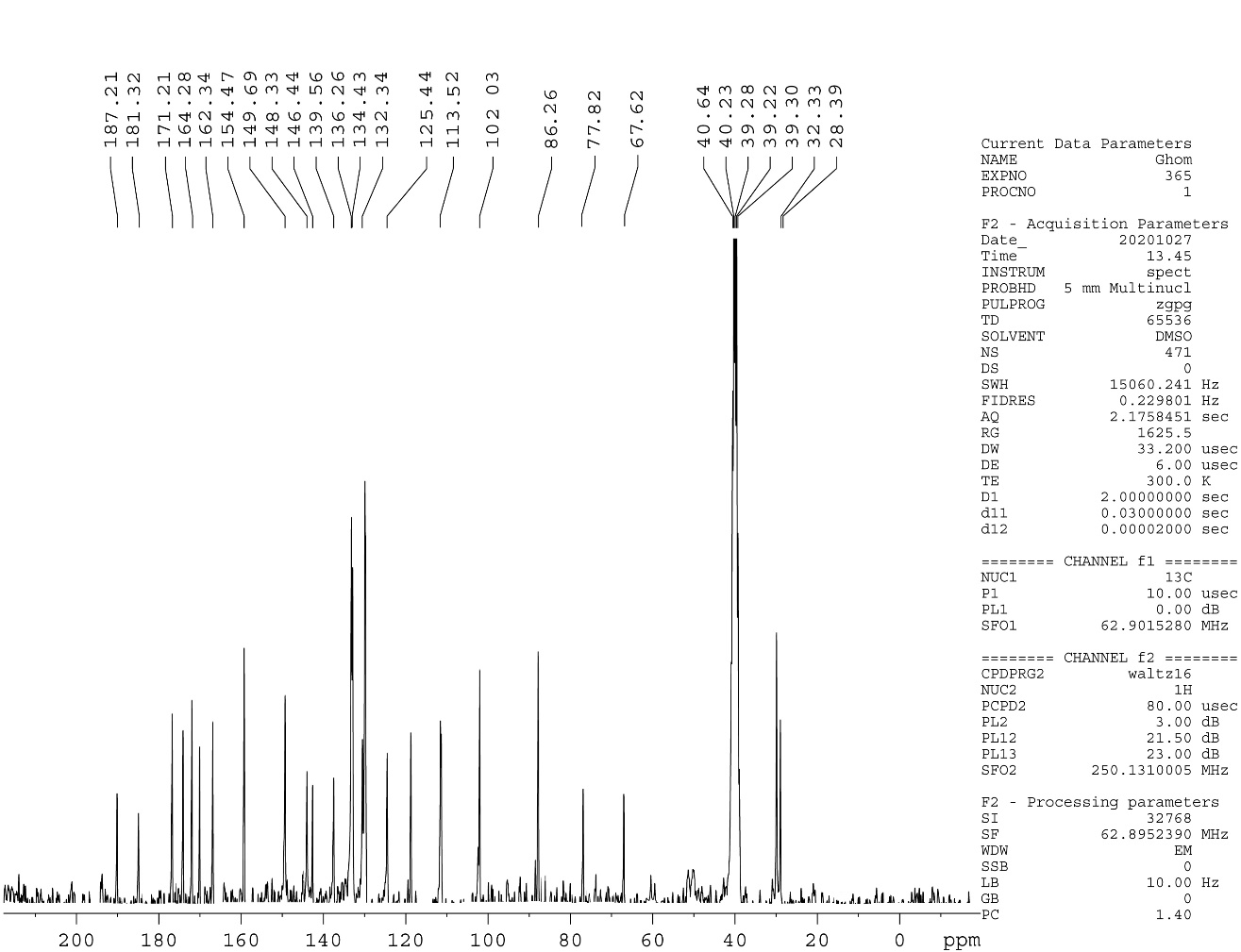
**

**Fig. S11.** ^13^C-NMR spectrum of 4k.

**2-amino-4-(4-cyanophenyl)-1-(4-methoxyphenyl)-7,7-dimethyl-5-oxo-1,4,5,6,7,8 hexahydroquinoline-3-carbonitrile 6c.** Yellow solid; m.p. 242-243°C. IR spectrum ν, cm^–1^: 3321, 3062, 2985, 2233, 1608, 1716, 1678, 1381, 1284; ^1^H NMR (250 MHz, DMSO-*d*_6_) δ: 0.82 (s, 3H, CH_3_), 0.95 (s, 3H, CH_3_), 2.29 (d, 2H, J = 8.4 Hz, 2CH), 2.44 (d, 2H, J = 8.2 Hz, 2CH), 3.82 (s, 3H, CH_3_), 4.33 (s, 1 H, CH), 5.33 (s, 2 H, NH_2_), 6.74-7.05 (d, 2H, J = 8.6 Hz, ArH), 7.28-7.32 (d, 2H, J = 8.3 Hz, ArH), 7.62-7.78 (m, 4H, ArH); ^13^C NMR (62.9 MHz, DMSO-*d*_6_) δ: 28.33, 30.79, 78.61, 88.62, 92.24, 102.06, 117.46, 118.41, 124.54, 128.61, 130.54, 132.93, 133.22, 137.51, 142.63, 144.01, 149.32, 159.27, 166.88, 173.46, 178.81, 180.74, 183.31, 192.12, 194.81, 196.71; Anal. Calcd. For: C_26_H_24_N_4_O_2_: C 73.56, H 5.70, N 13.20, O 7.54. Found: C 73.54, H 5.73, N 13.24, O 7.52; MS (EI) (m/z): 424.19 (M^+^).


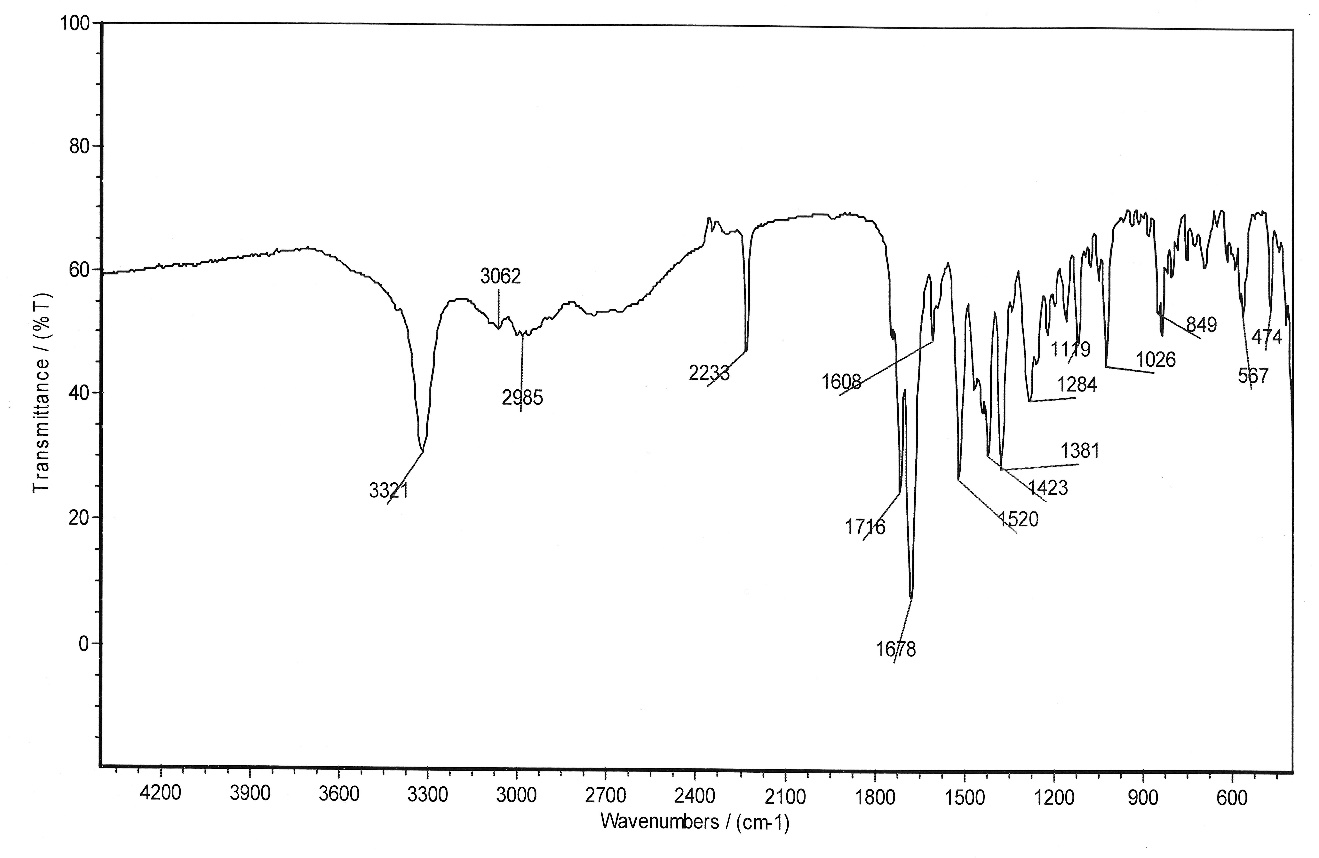


**Fig. S12.** FT-IR spectrum of 6c.


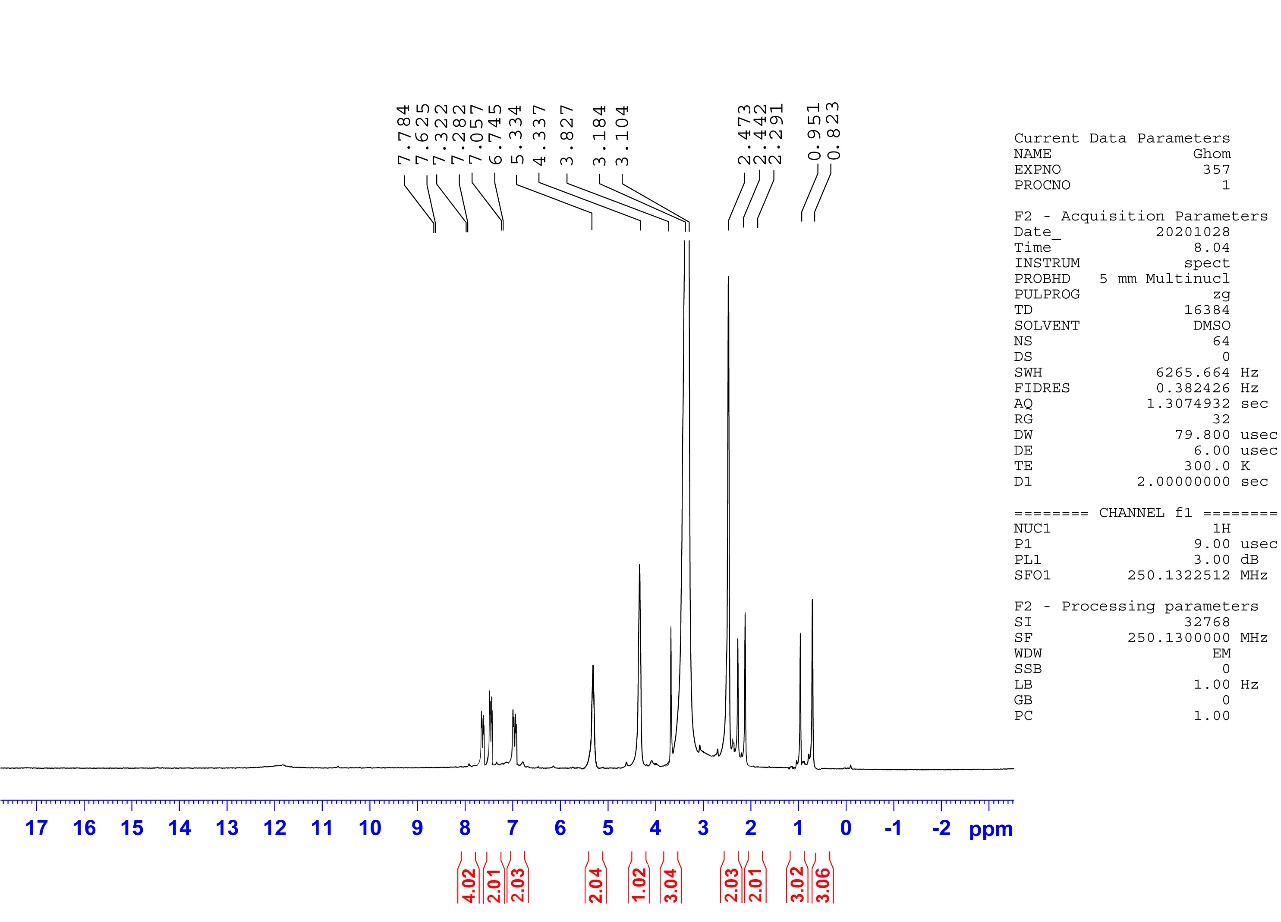


**Fig S13.** ^1^H-NMR spectrum of 6c.


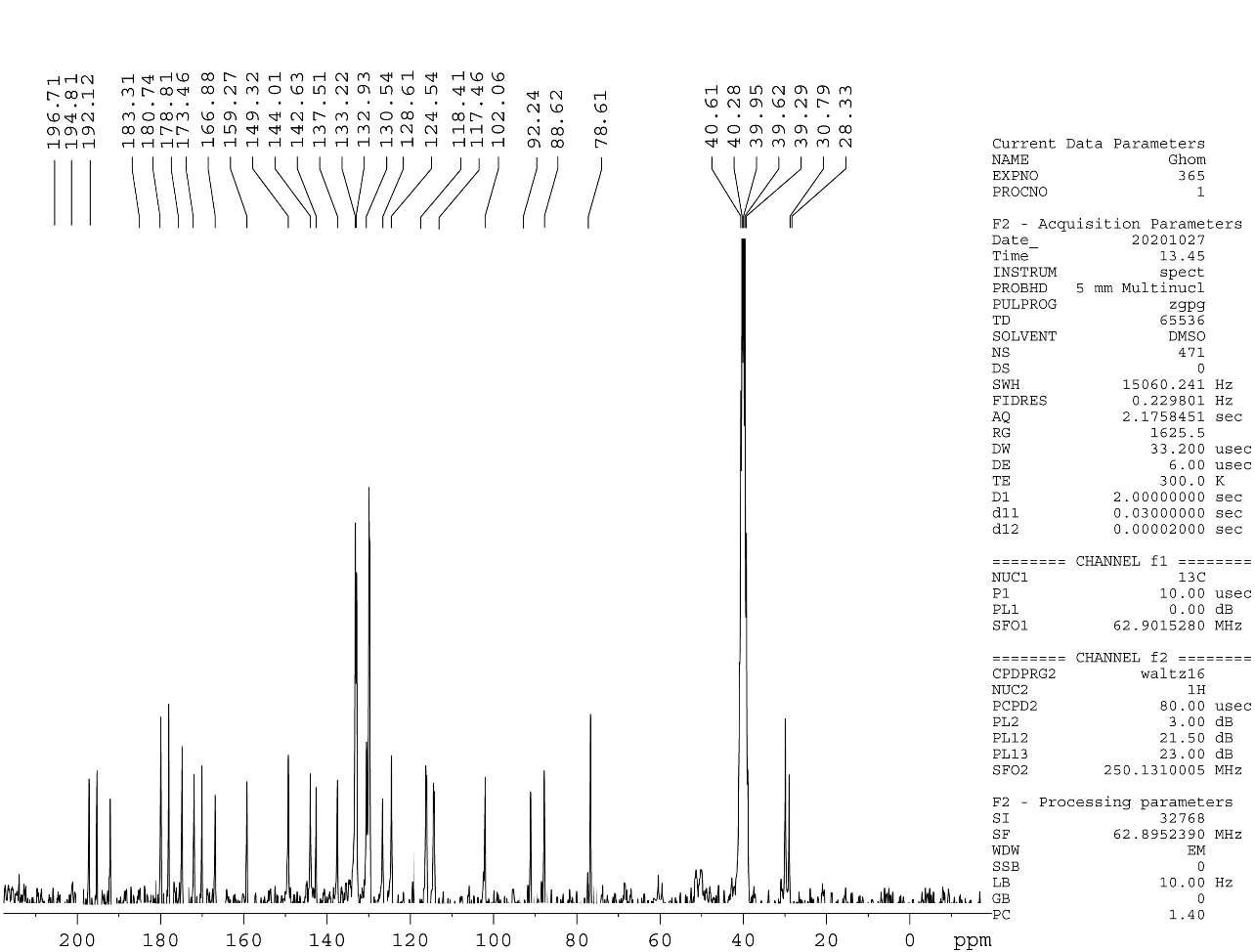


**Fig. S14.** ^13^C-NMR spectrum of 6c.

**2-amino-1-(4-methoxyphenyl)-7,7-dimethyl-4-(4-(methylthio)phenyl)-5-oxo-1,4,5,6,7,8 hexahydroquinoline-3-carbonitrile 6d.** Yellow solid; m.p. 256-258°C. IR spectrum ν, cm^–1^: 3352, 3186, 2962, 2191, 1685, 1651, 1604, 1369, 1211; ^1^H NMR (250 MHz, DMSO-*d*_6_) : 0.84 (s, 3H, CH_3_), 0.97 (s, 3H, CH_3_), 2.28 (d, 2H, J = 7.3 Hz, 2CH), 2.42 (d, 2H, J = 7.8 Hz, 2CH), 2.78 (s, 3H, SCH_3_), 3.83 (s, 3H, CH_3_), 4.32 (s, 1 H, CH), 5.33 (s, 2 H, NH_2_), 6.75-7.03 (d, 2H, J = 8.3 Hz ArH), 7.27-7.38 (d, 2H, J = 8.6 Hz, ArH), 7.64-7.75 (m, 4H, ArH); ^13^C NMR (62.9 MHz, DMSO-*d*_6_) δ: 27.33, 31.72, 76.26, 85.62, 94.22, 104.06, 116.41, 119.46, 123.54, 126.81, 131.54, 133.93, 135.22, 138.51, 141.62, 143.01, 148.32, 157.24, 164.87, 175.44, 176.86, 182.71, 185.31, 193.11, 193.82, 195.71; Anal. Calcd. For: C_26_H_27_N_3_O_2_S: C 70.09, H 6.11, N 9.43. O 7.18, S, 7.20. Found: C 70.06, H 6.16, N 9.48 O 7.12, S, 7.24; MS (EI) (m/z): 455.18 (M^+^).

**
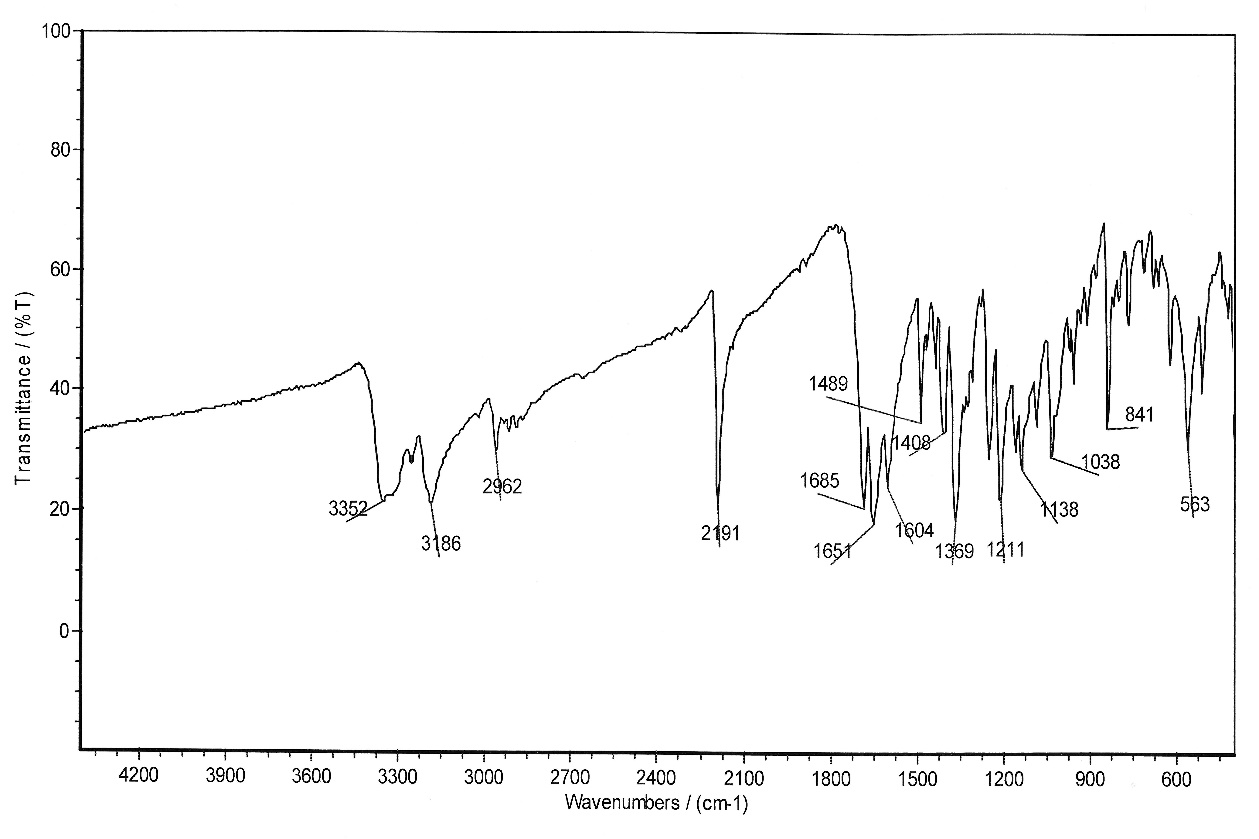
**

**Fig. S15.** FT-IR spectrum of 6d.


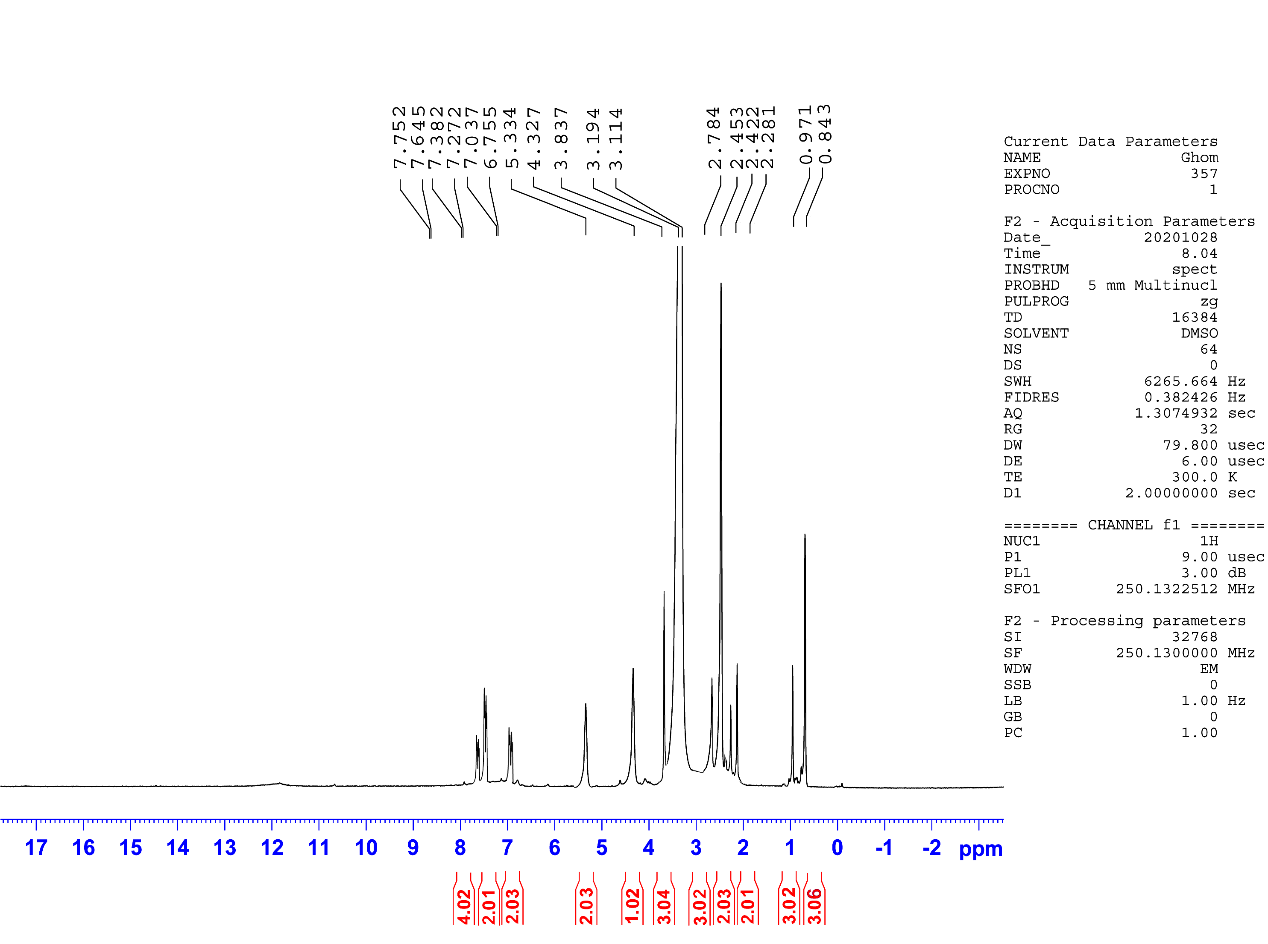


**Fig. S16.** ^1^H-NMR spectrum of 6d.


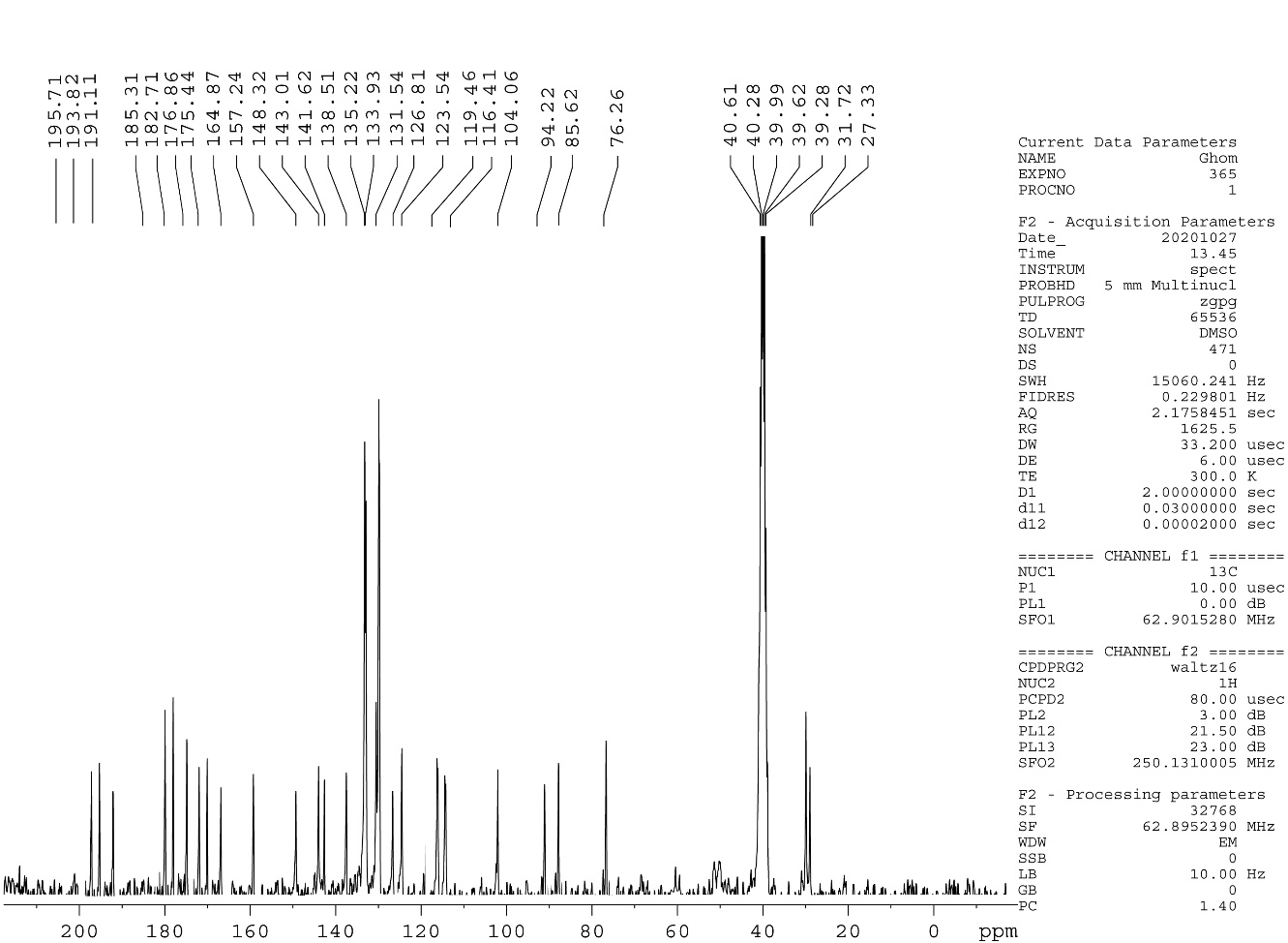


**Fig S17.** ^13^C-NMR spectrum of 6d.
